# Noncoding RNA regulates the expression of Krm1 and Dkk2 to synergistically affect aortic valve lesions

**DOI:** 10.1038/s12276-024-01256-5

**Published:** 2024-07-01

**Authors:** Gaopeng Xian, Rong Huang, Minhui Xu, Hengli Zhao, Xingbo Xu, Yangchao Chen, Hao Ren, Dingli Xu, Qingchun Zeng

**Affiliations:** 1 State Key Laboratory of Organ Failure Research, Department of Cardiology, Nanfang Hospital, Southern Medical University, 510515 Guangzhou, China; 2 Guangdong Provincial Key Laboratory of Cardiac Function and Microcirculation, Southern Medical University, 510515 Guangzhou, China; 3 Bioland Laboratory (Guangzhou Regenerative Medicine and Health Guangdong Laboratory), Guangzhou, China; 4 Department of Pharmacology and Experimental Therapeutics, Boston University School of Medicine, Boston, MA 02118 USA; 5 Department of Cardiology, University Medical Center of Goettingen, Robert-Koch-Str. 40, 37075 Goettingen, Germany; 6 School of Biomedical Sciences, Faculty of Medicine, The Chinese University of Hong Kong, Shatin, NT Hong Kong, China; 7 Department of Rheumatology, Nanfang Hospital, Southern Medical University, Guangzhou, China

**Keywords:** miRNAs, Valvular disease

## Abstract

Calcific aortic valve disease (CAVD) is becoming an increasingly important global medical problem, but effective pharmacological treatments are lacking. Noncoding RNAs play a pivotal role in the progression of cardiovascular diseases, but their relationship with CAVD remains unclear. Sequencing data revealed differential expression of many noncoding RNAs in normal and calcified aortic valves, with significant differences in circHIPK3 and miR-182-5p expression. Overexpression of circHIPK3 ameliorated aortic valve lesions in a CAVD mouse model. In vitro experiments demonstrated that circHIPK3 inhibits the osteogenic response of aortic valve interstitial cells. Mechanistically, DEAD-box helicase 5 (DDX5) recruits methyltransferase 3 (METTL3) to promote the N6-methyladenosine (m6A) modification of circHIPK3. Furthermore, m6A-modified circHIPK3 increases the stability of Kremen1 (Krm1) mRNA, and Krm1 is a negative regulator of the Wnt/β-catenin pathway. Additionally, miR-182-5p suppresses the expression of Dickkopf2 (Dkk2), the ligand of Krm1, and attenuates the Krm1-mediated inhibition of Wnt signaling. Activation of the Wnt signaling pathway significantly contributes to the promotion of aortic valve calcification. Our study describes the role of the Krm1-Dkk2 axis in inhibiting Wnt signaling in aortic valves and suggests that noncoding RNAs are upstream regulators of this process.

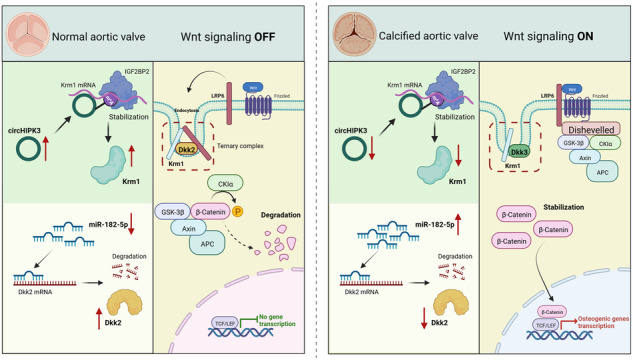

## Non-coding RNAs regulate aortic valve calcification via Wnt pathway

Calcific aortic valve disease (CAVD, a common heart condition) currently has no effective treatments. This research aimed to examine the role of non-coding RNAs (molecules that control gene activity) in CAVD, particularly circHIPK3 and miR-182-5p. Experiments were conducted on human heart valve cells and mice, showing that circHIPK3 can prevent heart valve hardening, while miR-182-5p can trigger a process that encourages hardening. The research also discovered a protein, Krm1, that can stop this hardening process. These results suggest that focusing on these non-coding RNAs and proteins could offer a new way to treat CAVD. However, more research is needed to fully comprehend these processes and their potential treatment implications.

This summary was initially drafted using artificial intelligence, then revised and fact-checked by the author.

## Introduction

Calcific aortic valve disease (CAVD) is a common acquired valve disease characterized by progressive thickening and functional stenosis of the aortic valve^1^. Between 1990 and 2017, more than 100,000 people died from CAVD^2^. However, there are no effective pharmacological therapies for preventing or reversing CAVD. Based on accumulating evidence, noncoding RNAs such as miRNAs and circRNAs play important roles in the development of cardiovascular diseases^3^. Adrenergic agonists induce oxoguanine (oG) modification at position 7 of miR-1 (7oG-miR-1), and 7oG-miR-1 accumulation leads to cardiac hypertrophy in mice^4^. Similarly, circ-INSR binds to single-stranded DNA binding protein (SSBP1) to prevent doxorubicin-mediated cardiotoxicity^5^. However, the regulatory role of noncoding RNAs in CAVD remains unclear. The most common cells in aortic valves are aortic valve interstitial cells (AVICs), whose phenotypic switch to an osteoblast-like phenotype is thought to be the pathological cause of aortic valve calcification^6^. Studies have shown that Wnt signaling is a critical driver of osteogenesis. Moreover, the function of Wnt signaling in inducing osteogenic responses and the development of aortic valve calcification has been shown in several in vitro and animal studies^7,8^. Importantly, miR-138-5p was recently shown to suppress the osteogenic response of AVICs by inhibiting the Wnt/β-catenin pathway^9^. Kremen1, also known as Krm1, is a transmembrane protein involved in Dickkopf (Dkk)-mediated antagonism of canonical Wnt signaling^10^. Canonical Wnt signaling activation depends on the formation of a ternary complex of Wnt ligands with Frizzled receptors and LDL receptor-related protein 5/6 (LRP5/6), which promotes β-catenin stabilization and nuclear translocation and subsequently activates pro-osteogenic gene expression^11^. However, Dkks form a three-component complex with Krm1 and LRP5/6, which is then rapidly endocytosed and removed from the membrane, blocking Wnt signaling^11^. In *Xenopus*, Krm1 cooperates with Dkks to inhibit the Wnt pathway and regulate the early anteroposterior pattern of the central nervous system^12^. Therefore, targeting Krm1 and Dkks to inhibit Wnt pathway activation may be an effective approach for regulating CAVD, but the importance of Dkks and Krm1 in CAVD has not been reported.

N6-methyladenosine (m6A) modifications are the most abundant base modifications in eukaryotic cells and are involved in important biological processes such as RNA splicing, degradation, and translation^13^. Interestingly, recent studies have shown that m6A-modified circRNAs increase the stability of target mRNAs^14,15^. In this study, circHIPK3 promoted Krm1 expression in an m6A-dependent manner. In addition, miR-182-5p is synergistically involved in the regulation of aortic valve lesions by modulating Dkk2 expression. The present study aimed to elucidate the intrinsic mechanism by which noncoding RNAs synergistically modulate aortic valve lesions by regulating Krm1-Dkk2 interactions.

## Materials and methods

### Human aortic valve collection

Normal human aortic valve leaflets were collected from the explanted hearts of patients undergoing heart transplantation, and calcified aortic valve leaflets were obtained from patients with CAVD who were undergoing aortic valve replacement. All patients provided informed consent for the use of their valves in this study. For this study, the protocol was performed in compliance with the Declaration of Helsinki and was approved by Nanfang Hospital, Southern Medical University. The baseline characteristics of the non-CAVD patients and CAVD patients are shown in Supplementary Table 1.

### Isolation and culture of human AVICs

Primary human AVICs were isolated and cultured by using a previously described method^16^. Briefly, fresh aortic valve leaflets were washed several times with Hank’s solution on an ultraclean table and then digested with collagenase I, after which the cells were collected by centrifugation. The AVICs were cultured in M199 growth medium supplemented with penicillin G, streptomycin, amphotericin B, and 10% fetal bovine serum, and the medium was changed every three days. When the cells reached 80–90% confluence, they were subcultured on plates. AVICs from passages 3–6 were used in this study.

### RNA-seq

RNA-seq was performed by Cloud Sequencing (Shanghai, China), which provided the final sequencing data and analysis. Total RNA was isolated from the tissue samples using TRIzol reagent (Life Technologies, Carlsbad, CA, USA) according to the manufacturer’s instructions. Before construction of the RNA-seq libraries, the Ribo-Zero rRNA Removal Kit (Illumina, San Diego, CA, USA) and the CircRNA Enrichment Kit (Cloud-seq, Shanghai, China) were used to remove the rRNA and enrich the circRNAs. The RNA-seq libraries were constructed by using pretreated RNAs with a TruSeq Stranded Total RNA Library Prep Kit (Illumina, San Diego, CA, USA) according to the manufacturer’s instructions, and the libraries were sequenced on an Illumina HiSeq™ 4000 Sequencer (Illumina, San Diego, CA, USA). The CircBase database was used to annotate the identified circRNAs. All detected circRNAs and differentially expressed circRNAs are listed in the Excel spreadsheet (Supplementary Material 2 and GSE254393, Supplementary Material 3).

### Fluorescence in situ hybridization

A fluorescence in situ hybridization (FISH) kit (lnc1101036, RiboBio, Guangzhou, China) was used according to the manufacturer’s instructions. Briefly, AVICs were cultured in 15 mm confocal dishes. Then, the cells were washed with phosphate-buffered saline (PBS), fixed with 4% paraformaldehyde, permeabilized with 0.5% Triton X-100, and incubated overnight at 37 °C with RNA probes in hybridization buffer. Subsequently, the cells were rinsed with 2× saline sodium citrate (SSC), and the cell nuclei were stained with DAPI solution. Finally, the specimens were analyzed using a Leica confocal fluorescence microscope (STELLARIS 5, Leica, Germany).

### Western blotting

Equal amounts of protein lysates were resolved on SDS‒PAGE gels, and the proteins were then transferred to PVDF membranes (Millipore, USA). After incubation with a primary antibody at 4 °C overnight, the membranes were hybridized with a secondary antibody at room temperature for 1 h. The immunoreactive signals were visualized by an enhanced chemiluminescence kit (Fdbio, China). Primary antibodies against the following proteins were used: ALP (ab83259, Abcam), BMP-2 (ab14933, Abcam), RUNX2 (ab76956, Abcam), Krm1 (#9592, CST), LRP6 (#2560 S, CST), YTHDF2 (24744-1-AP, Proteintech), IGF2BP2 (ab124930, Abcam), DDX5 (ab126730, Abcam), METTL3 (15073-1-AP, Proteintech), and β-catenin (#8480 S, CST). We used GAPDH (ab181602, Abcam), ATP1A1 (14418-1-AP, Proteintech), and β-actin (81115-1-RR, Proteintech) antibodies as controls. HRP-conjugated goat anti-mouse IgG (FDM007, 1:5000 dilution; Fdbio, China) and HRP-conjugated goat anti-rabbit IgG (FDR007, 1:5000 dilution; Fdbio, China) were used as the secondary antibodies.

### RNA quantification

Total RNA from cell or tissue lysates was isolated using RNAiso Plus (TaKaRa, Dalian, China). Reverse transcription was performed using PrimeScript RT Master Mix (TaKaRa, Dalian, China). All qRT‒PCR analyses were performed using TB Green PCR Master Mix (TaKaRa, Dalian, China) in a LightCycler 480 System (Roche, Germany). Briefly, 1 µl of cDNA, 5 µl of TB Green Premix, 0.4 µl of forward and reverse primers (10 µM), and 3.2 µl of RNase-free water were used in a 10 µl reaction system. The reaction mixture was added to a 96-well PCR plate and placed in a LightCycler 480 system for amplification. The mRNA levels were calculated for each sample using the cycle threshold (Ct) and ∆CT methods, with GAPDH serving as an endogenous control. The relative fold difference between groups was calculated using the 2-∆∆CT method.

For miRNAs, we used tailing reaction analysis. Briefly, the RNA molecule was polyadenylated and then reverse transcribed using an mRQ enzyme mixture, which included poly(A) polymerase and SMART MMLV RT (Cat. #638316, TaKaRa, Dalian, China). Subsequently, the target miRNA was specifically detected by qPCR (Cat. #638316, TaKaRa, Dalian, China). The primers used for reverse transcription and the reverse primers used for qPCR were obtained from the kit.

### Agarose gel electrophoresis

Agarose gels (2%) were prepared by melting two grams of RNase-free agarose per 100 ml of TAE running buffer and pouring them into casting trays. After cooling and solidification, the PCR products were loaded into each well, with the iVDye 100 bp DNA Ladder (V1002-025, Gendepot, USA) serving as a marker. Electrophoresis was performed at a constant voltage for approximately 30 min, and then, Bio-Rad Gel Doc XR (Bio-Rad Laboratories, Hercules, CA, USA) was used to capture images.

### Overexpression and knockdown of genes

For siRNA experiments, the following siRNAs were used: human circHIPK3 siRNA (RiboBio, Guangzhou, China), human DDX5 siRNA, human METTL3 siRNA, human YTHDF2 siRNA, human Krm1 siRNA, and human Dkk2 siRNA, while Scr siRNA was used as a control. These siRNAs were all provided by Thermo Fisher Scientific (Supplementary Material 1). Transient transfection with siRNA was performed using Lipofectamine 3000 (Thermo Fisher Scientific, USA), and siRNA was reverse-transfected into cells according to the supplied protocol. Briefly, Lipofectamine 3000 and siRNA (20 nM) were mixed with Opti-MEM (Thermo Fisher Scientific, USA) at 70% confluence, incubated at room temperature for 15 min, and then added to the cell culture dish, and the medium was changed after 6‒8 h.

For overexpression experiments, according to the instructions, Lipofectamine 3000, P3000 Reagent (2 µl/µg), and plasmids (5 µg) were mixed with Opti-MEM, incubated at room temperature for 15 min, and then added to a 6-well cell culture dish; the medium was changed after 6‒8 h.

### RNase R treatment

RNase R treatment was performed as described previously. Briefly, 2 µg of total RNA was incubated with or without 3 U/µg RNase R (Sigma, USA) at 37 °C for 30 min. The RNA expression levels of circHIPK3 and linear HIPK3 were analyzed using qRT‒PCR.

### Transfection of miRNA mimic or inhibitor

Lipofectamine 3000 was used to transfect miR-182-5p mimic, miR-NC mimic, miR-182-5p inhibitor, and miR-NC inhibitor into AVICs. Briefly, Lipofectamine 3000 and mimic or inhibitor (20 nM) were mixed with Opti-MEM, incubated at room temperature for 15 min, and then added to the cell culture dish; the medium was changed after 6‒8 h.

### Luciferase reporter assay

HEK-293T cells were seeded in 96-well plates and cultured to 50%–70% confluence before transfection. For Dkk2 and miR-182-5p, either wild-type or mutant Dkk2 3′UTR fragments were inserted into the psi-CHECK2-firefly luciferase vector (Promega, USA). Dkk2 3′UTR-WT plasmids, Dkk2 3′UTR-MUT plasmids, miR-182-5p mimics, miR-NC, miR-182-5p inhibitors, and miR-NC inhibitors were transfected into cells. After 48 h of incubation, the Promega Dual-Luciferase system was used to assess firefly and Renilla luciferase activities.

### Immunofluorescence staining

Cultured cells were fixed in 4% paraformaldehyde for 15 min, permeabilized with 0.5% Triton in PBS for 10 min, and incubated with 5% goat serum for 1 h at room temperature. The cells were then incubated with the primary antibody at 4 °C overnight. After PBS washes, the slides were incubated with goat anti-mouse IgG H&L (Alexa Fluor® 647, ab150115, Abcam) or goat anti-rabbit IgG H&L (Alexa Fluor® 488, ab150077, Abcam) for 1 h at room temperature. Finally, the nuclei were stained with Hoechst dye. Images were obtained with a Leica confocal microscope.

For mouse aortic valve tissue, paraffin-embedded sections were prepared, dewaxed in xylene, and rehydrated in graded ethanol. Then, the sections were heated in 10 mM citrate buffer (pH 6.0) for antigen retrieval. The slides were incubated with 5% goat serum for 1 h at room temperature and then incubated with primary antibodies overnight at 4 °C. After PBS washes, the slides were incubated with goat anti-mouse IgG H&L and anti-WGA (ab178444, Abcam) for 1 h at room temperature. Finally, the nuclei were stained with Hoechst dye. Images were obtained with a Leica confocal microscope.

### H&E staining and von Kossa staining

Aortic valves from animals were embedded in paraffin and then cut into 5-μm-thick sections. Sections were stained with an H&E staining kit (G1120, Solarbio, China) and a Von Kossa staining kit (G1043, Servicebio, China) to measure aortic valve thickness and calcium deposits. Briefly, paraffin-embedded sections were prepared, dewaxed in xylene, and rehydrated in graded ethanol. The tissue sections were then incubated with the corresponding dye solution, washed in water to remove excess dye, and finally examined and photographed with an Olympus CKX41 microscope (Olympus, Japan).

### Alizarin red staining

AVICs were cultured in osteogenic medium (M199 medium supplemented with 10 mmol/L β-glycerophosphate, 10 nmol/L dexamethasone, 4 μg/ml cholecalciferol and 8 mmol/L CaCl_2_) for 14–21 days, washed twice with PBS and fixed for 15 min in 4% paraformaldehyde. After 30 min of incubation with 0.2% alizarin red solution (pH 4.0–4.2), the excess dye was removed by washing with distilled water, and the samples were finally examined and photographed with an Olympus CKX41 microscope.

### ALP activity

The cells were fixed, and histochemical staining for ALP activity was performed as previously described^17^. Briefly, cell monolayers were washed with PBS and fixed for 10 min in 4% paraformaldehyde, followed by incubation with a mixture of 0.1 mg/ml naphthol AS-MX phosphate, 0.5% N,N-dimethylformamide, 2 mM MgCl_2_, and 0.6 mg/ml fast blue BB salt in 0.1 M Tris-HCl, pH 8.5, at room temperature for 30 min. Excess dye was removed by washing the sections with PBS, and the sections were finally examined and photographed with an Olympus CKX41 microscope.

### Nuclear and cytoplasmic fraction extraction

The nuclear and cytoplasmic protein components of AVICs were isolated using NE-PER nuclear and cytoplasmic extraction reagents (Thermo Fisher Scientific, USA) according to the manufacturer’s protocol. Briefly, cells were harvested using trypsin-EDTA and then centrifuged at 500 × *g* for 5 min. Subsequently, the cells were washed once with PBS. Next, ice-cold CER I was added to the cell pellet, which was thoroughly mixed and incubated on ice for 10 min. Ice-cold CER II was then added to the tube, followed by incubation on ice for 1 min. Finally, the tube was centrifuged at 16,000 × *g* for 5 min in a microcentrifuge. The resulting supernatant represented the cytoplasmic extract, while the pellet contained the nuclear fraction. GAPDH and U6 served as controls for the cytoplasmic and nuclear fractions, respectively.

### Coimmunoprecipitation (Co-IP)

We used IP lysis buffer (Invitrogen, Carlsbad, CA, USA) to lyse the cells. The cell lysates were incubated with specific antibodies for 4 h and then added to Protein A/G Sepharose beads (Santa Cruz CA, United States), which were rotated overnight at 4 °C. With the help of magnetic support, the beads were washed three times with PBS and resuspended in SDS‒PAGE loading buffer for western blot analysis using the corresponding antibodies. Primary antibodies against the following proteins were used: Krm1 (#9592, CST), LRP6 (#2560 S, CST), DDX5 (ab126730, Abcam), and METTL3 (15073-1-AP, Proteintech).

### RNA immunoprecipitation (RIP)

The RIP assay was performed by using an RNA Immunoprecipitation Kit (Geneseed, Guangzhou, China) according to the manufacturer’s instructions. Briefly, the treated AVICs were collected and lysed in RIP lysis buffer containing a protease inhibitor cocktail and RNase inhibitor on ice for 30 min. After centrifugation, the supernatant was incubated with 30 μL of Protein A/G Sepharose beads and antibodies. After overnight incubation at 4 °C, the immunocomplexes were centrifuged and then washed six times with washing buffer. The bead-bound proteins were further analyzed by western blotting. The immunoprecipitated RNA was subjected to qRT‒PCR analysis. Antibodies against DDX5 (ab126730, Abcam) and IGF2BP2 (ab124930, Abcam) were used to precipitate RNA.

### Methylated RNA immunoprecipitation (MeRIP)

MeRIP was performed using a BersinBio™ MeRIP Kit (BersinBio, Guangzhou, China). Briefly, we extracted total RNA from the AVICs and used RNase R to digest the linear RNA, after which the digestion-resistant circRNA was purified and retained. Then, the lysate was immunoprecipitated with an anti-m6A antibody (A17924, ABclonal, China) overnight at 4 °C. The immunoprecipitated RNA was subjected to qRT‒PCR analysis, and the amplification of a single product was confirmed by agarose gel visualization and/or melting curve analysis.

### Animal models

Animal experiments were conducted in accordance with the guidelines of Directive 2010/63/EU of the European Parliament on the protection of animals used for scientific purposes. This study was approved by the Nanfang Hospital Animal Ethics Committee. In this study, C57BL/6 mice and ApoE^−^^/^^−^ mice were used to establish CAVD mouse models (Supplementary Table 2).

Eight-week-old mice received tail vein injections of adeno-associated virus subtype 2 expressing circHIPK3 (Vigene Biosciences, Shandong, China), while empty adeno-associated virus was used as a control. The mice received tail vein injections of virus every 4 weeks until the end of the experiment. Injury and high-fat diet feeding were performed 4 weeks after the first injection. Twelve-week-old C57BL/6 mice with wire-injured heart valves were established as previously described^18^. Briefly, the right carotid artery was exposed by blunt dissection, and a wire was introduced into the artery. The wire was slowly rotated and carefully inserted into the left ventricle under ultrasound guidance. Aortic valve injury was induced by scratching the leaflets with the body of the wire. A sham procedure was performed in the same manner without insertion of the wire into the left ventricle. An High-fat diet (HFD) was fed to twelve-week-old ApoE^−^^/^^−^ mice to induce aortic valve calcification^19^.

After 24 weeks, we employed Vevo 2100 ultrasound (Visual Sonics, Toronto, ON, Canada) after inhalation anesthesia with 2% isoflurane to assess the morphology of the aortic valve and the function of the heart. Finally, the mice were euthanized by intraperitoneal injection of sodium pentobarbital (200 mg/kg), and the hearts were collected.

### Statistics

All the statistical analyses were performed using GraphPad Prism software (version 9.0). Each experiment was repeated at least three times. All the data are presented as the means ± SDs. The differences between experimental groups were evaluated using two-tailed Student’s *t*-test (2-group comparisons) or one-way or two-way repeated measures ANOVA followed by a Bonferroni post hoc correction (multigroup comparisons) as appropriate. For time-course data, two-way repeated measures ANOVA was used to compare differences between experimental groups at each time point. A value of *P* < 0.05 indicated statistical significance.

## Results

### CircHIPK3 expression is decreased in calcified aortic valves

**Fig. 1:**
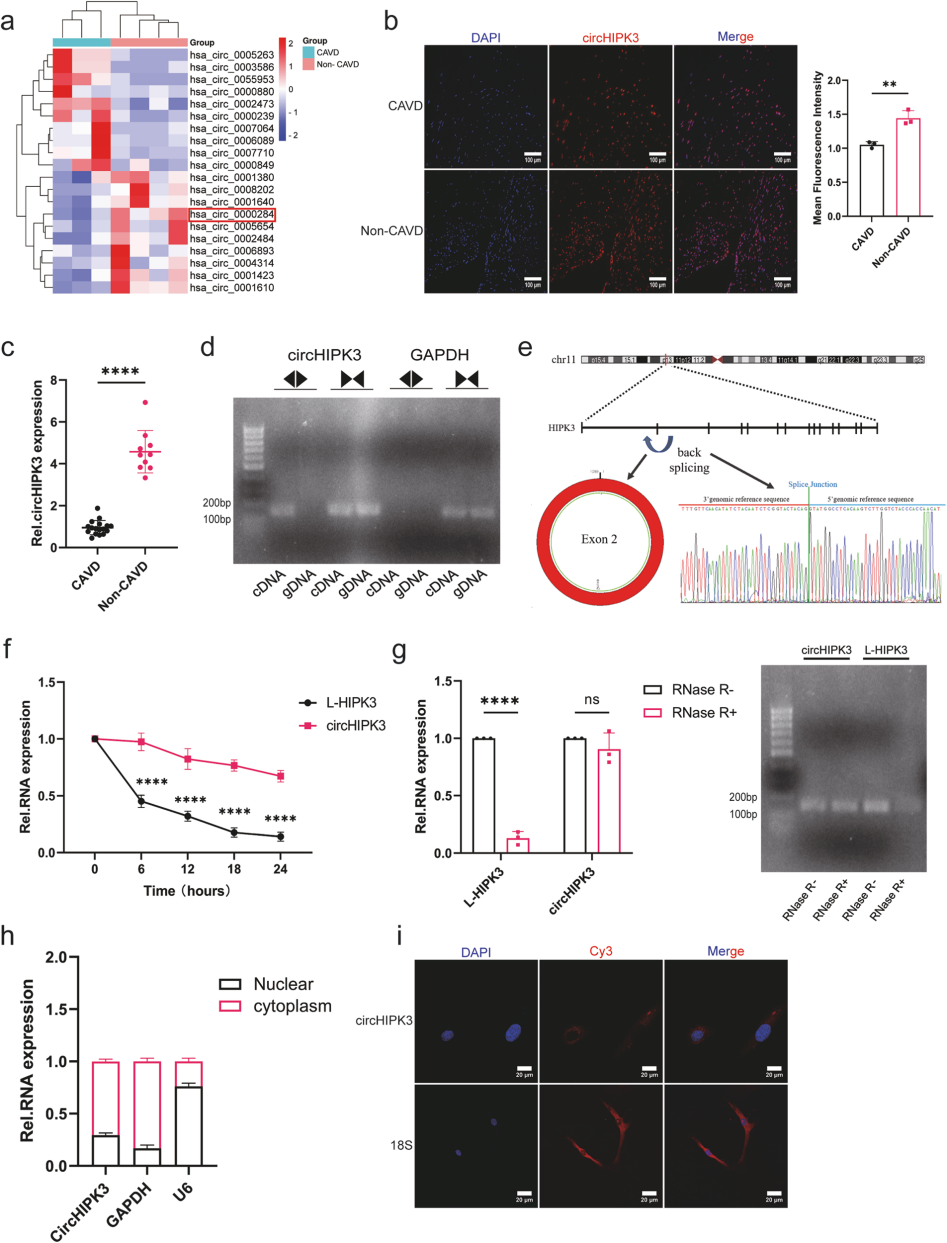
CircHIPK3 is a circRNA associated with CAVD. **a** Three calcified human aortic valves and four noncalcified human aortic valves were subjected to high-throughput sequencing, and circHIPK3 was found to be abundantly expressed in aortic valves, but its expression was decreased in calcified aortic valves. **b** Distribution of circHIPK3 in the aortic valve, as determined using RNA FISH (*n* = 3, bar = 100 μm). **c** qRT‒PCR was used to quantify the expression of circHIPK3 in calcified and noncalcified aortic valves (calcified = 17, noncalcified = 10). The error bars show the means ± SDs. *P*-values were determined by a two-tailed Student’s *t*-test. **d** Divergent primers were used to amplify circHIPK3 from cDNA instead of from genomic DNA (gDNA). The direction of the arrow indicates the divergent and convergent primers. GAPDH was used as a negative control. **e** Sanger sequencing confirmed that the sequence of circHIPK3 was consistent with the known sequence. **f** Detection of circHIPK3 and linear HIPK3 (L-HIPK3) expression in AVICs treated with actinomycin D at the designated time points (*n* = 3, ANOVA followed by Bonferroni post hoc correction). **g** Total RNA was digested with RNase R, followed by qRT‒PCR detection of circHIPK3 and L-HIPK3 expression (*n* = 3, ANOVA followed by Bonferroni post hoc correction). The right panel shows the results of agarose gel electrophoresis. **h** qRT‒PCR demonstrated that circHIPK3 mainly exists in the cytoplasm of AVICs. GAPDH was used as a cytoplasmic marker, and U6 was used as a nuclear marker (*n* = 3). **i** RNA FISH was used to explore the localization of circHIPK3 in AVICs. 18S RNA was used as a cytoplasmic marker (*n* = 3, bar = 20 µm). Asterisks represent statistically significant differences (**P < 0.01, ***P < 0.001, and ****P < 0.0001).

For analysis of the expression of circRNAs in the aortic valve, we performed high-throughput sequencing of calcified and noncalcified human aortic valves and identified circRNAs by reading junction sites (Supplementary Material 2, GSE254393). A total of 19,832 circRNAs were identified, 140 of which were differentially expressed in human aortic valves (Fig. 1a). Among these 140 differentially expressed circRNAs, hsa_circ_0000284 (circHIPK3) exhibited the highest basal expression level, indicating that this molecule showed the most prominent change. Additionally, research has shown that circHIPK3 plays a crucial role in cardiovascular diseases^20^ and can inhibit vascular calcification^21^. Therefore, we selected circHIPK3 as the target circRNA of our study. Next, fluorescence in situ hybridization (FISH) and qRT‒PCR analysis revealed decreased expression of circHIPK3 in human calcified aortic valves, consistent with the sequencing results (Fig. 1b, c). Additionally, we amplified circHIPK3 from cDNA but not from genomic DNA (gDNA) using divergent primers, suggesting that this RNA species is circular (Fig. 1d). The Sanger sequencing results were consistent with the circHIPK3 sequence obtained from the circBase database^22^ (Fig. 1e). After treatment with actinomycin D (a transcriptional inhibitor), qRT‒PCR revealed that the half-life of circHIPK3 was longer than that of linear HIPK3 (L-HIPK3) (Fig. 1f). Similarly, compared with L-HIPK3, circHIPK3 was more resistant to ribonuclease R (RNase R) digestion (Fig. 1g). Furthermore, nuclear and cytoplasmic RNA isolation for qRT‒PCR (Fig. 1h, Supplementary Fig. 1a) and FISH experiments (Fig. 1i) showed that circHIPK3 was primarily located in the cytoplasm of aortic valve interstitial cells (AVICs).

### CircHIPK3 suppresses aortic valve calcification in vivo

**Fig. 2:**
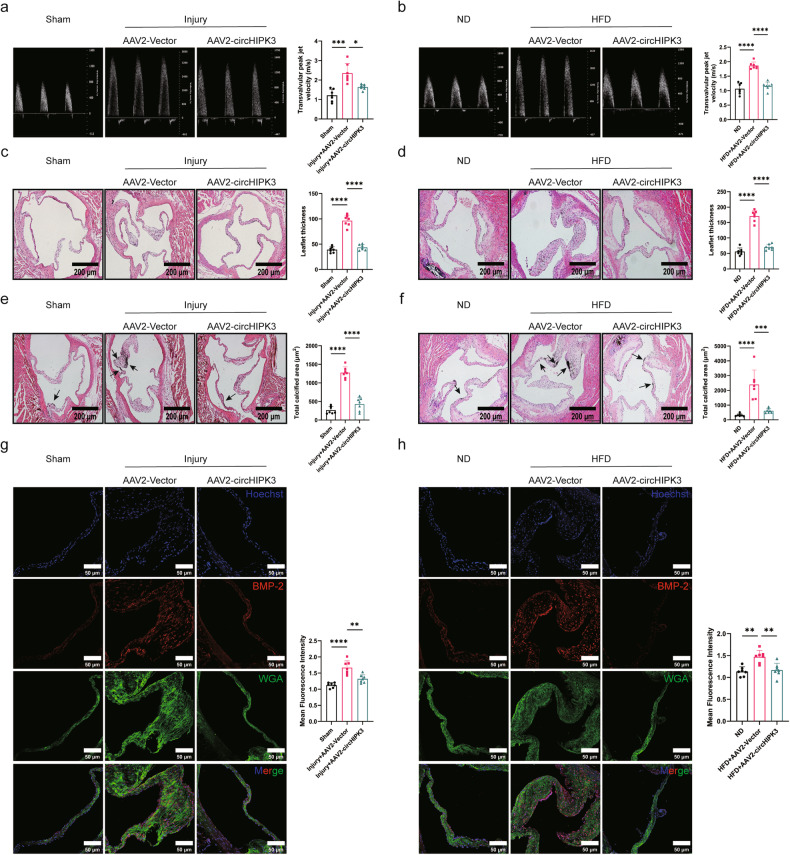
CircHIPK3 alleviates aortic valve calcification in vivo. **a**, **b** The transvalvular peak jet velocity was measured in C57BL/6 mice and ApoE^−^^/^^−^ mice from different groups (*n* = 7). **c**, **d** Analysis of aortic valve leaflet morphology using H&E staining in C57BL/6 mice and ApoE^−^^/^^−^ mice from different groups (*n* = 7). **e**, **f** Analysis of calcium deposits using von Kossa staining in C57BL/6 mice and ApoE^−^^/^^−^ mice from different groups (*n* = 7). Areas of calcium deposition are indicated by arrows. **g**, **h** Protein levels of the osteogenic marker BMP-2 in C57BL/6 mice and ApoE^−^^/^^−^ mice from different groups were determined by immunofluorescence staining (*n* = 7, bar = 50 μm). All the data are shown as the mean ± SD. *P*-values were determined by ANOVA followed by Bonferroni post hoc correction. Asterisks represent statistically significant differences (**P < 0.01, ***P < 0.001, and ****P < 0.0001).

In this study, CAVD mouse models were established by direct wire injury to the aortic valve in C57BL/6 mice^18^ and feeding ApoE^−/−^ mice a high-fat diet (HFD)^19^. To investigate the therapeutic potential of circHIPK3 in aortic valve calcification, we overexpressed circHIPK3 in vivo by injecting adeno-associated virus subtype 2 expressing circHIPK3 (AAV2-circHIPK3) through the tail vein of the mice, while the empty AAV2 vector (AAV2-vector) served as a control. The circHIPK3 overexpression in the aortic valve was verified (Supplementary Fig. 2a, b). After 24 weeks, echocardiography revealed a significant increase in the transvalvular peak jet velocity (Fig. 2a, b) and a decrease in the aortic valve area (AVA) in the CAVD model mice (Supplementary Fig. 2c, d). This finding suggested that the mice with CAVD have aortic valve stenosis. Furthermore, H&E staining and von Kossa staining revealed increased thickness and calcium deposition in the aortic valve leaflets (Fig. 2c–f). Immunofluorescence staining revealed that the expression of bone morphogenetic protein 2 (BMP-2), a key osteogenic marker, was significantly upregulated in the mice with CAVD (Fig. 2g, h). These results strongly suggest that calcification occurs in the aortic valve of mice with CAVD. Excitingly, injection of AAV2-circHIPK3 in the CAVD model mice alleviated aortic valve stenosis, decreased thickness and calcium deposition in the aortic valve leaflets, and downregulated BMP-2 expression. These results demonstrated that circHIPK3 attenuated aortic valve calcification in vivo.

### CircHIPK3 inhibits the osteogenic response of AVICs in vitro

**Fig. 3:**
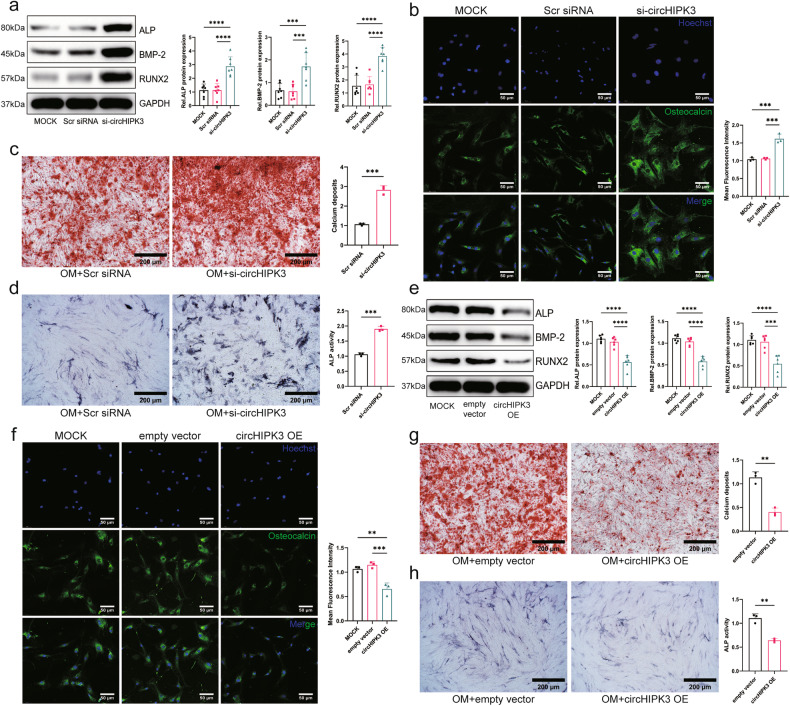
CircHIPK3 inhibits the osteogenic responses of AVICs in vitro. **a** Western blot analysis of the expression of osteogenic markers (ALP, BMP-2, and Runx2) after transfection with scrambled siRNA or circHIPK3 siRNA (*n* = 4, ANOVA followed by a Bonferroni post hoc correction). **b** Osteocalcin expression in AVICs transfected with scrambled siRNA or circHIPK3 siRNA was determined by immunofluorescence staining (*n* = 3, bar = 50 μm, ANOVA followed by a Bonferroni post hoc correction). **c** AVICs were cultured with osteogenic medium (OM) for 14–21 days, transfected with scrambled siRNA or circHIPK3 siRNA, and incubated with 0.2% alizarin red solution for 30 min, after which calcium deposits were observed with an inverted microscope (*n* = 3, bar = 200 μm, two-tailed Student’s *t*-test). The red area represents calcium nodules. **d** ALP activity was observed in AVICs transfected with scrambled siRNA or circHIPK3 siRNA upon OM induction (*n* = 3, bar = 200 μm, two-tailed Student’s *t*-test). Areas of active ALP activity are indicated in blue. **e** The protein levels of osteogenic markers were measured after the transfection of AVICs with the vector or the circHIPK3 overexpression vector (*n* = 6, ANOVA followed by a Bonferroni post hoc correction). **f** Immunofluorescence staining was used to assess osteocalcin expression in the AVICs transfected with the vector or the circHIPK3 overexpression vector (*n* = 3, bar = 50 μm, ANOVA followed by a Bonferroni post hoc correction). **g** Analysis of calcium deposition in the AVICs transfected with the vector or circHIPK3 overexpression vector using alizarin red S staining (*n* = 3, bar = 200 μm, two-tailed Student’s *t*-test). The red area represents the formation of calcium nodules. **h** The ALP activity of the AVICs treated with the vector or the circHIPK3 overexpression vector was determined (*n* = 3, bar = 200 μm, two-tailed Student’s *t*-test). Areas of active ALP activity are indicated in blue. Asterisks represent statistically significant differences (**P < 0.01, ***P < 0.001, and ****P < 0.0001).

Since the phenotypic switching of AVICs to an osteoblast-like phenotype is an important cause of aortic valve calcification, we further investigated whether circHIPK3 affects the osteogenic response of AVICs. We designed a specific small interfering RNA (siRNA) based on the backsplicing site of circHIPK3 to inhibit circHIPK3 expression without affecting L-HIPK3 expression (Supplementary Fig. 3a). Notably, after knockdown of circHIPK3, the expression of osteogenic markers such as alkaline phosphatase (ALP), BMP-2, Runt-related transcription factor 2 (Runx2), and osteocalcin was significantly increased in AVICs (Fig. 3a, b). Calcium deposition and ALP activity in AVICs were increased after circHIPK3 knockdown (Fig. 3c, d). To further investigate the role of circHIPK3 in the osteogenic response of AVICs, we elevated circHIPK3 expression in AVICs by transfecting these cells with a circHIPK3 overexpression plasmid (circHIPK3 OE) using an empty vector as a control (Supplementary Fig. 3b). As expected, the expression of osteogenic markers, calcium deposition, and ALP activity were significantly decreased in AVICs after overexpression of circHIPK3 (Fig. 3e–h). These results indicated that circHIPK3 inhibited the osteogenic response of AVICs.

### CricHIPK3 promotes Krm1 expression in an m6A-dependent manner

**Fig. 4:**
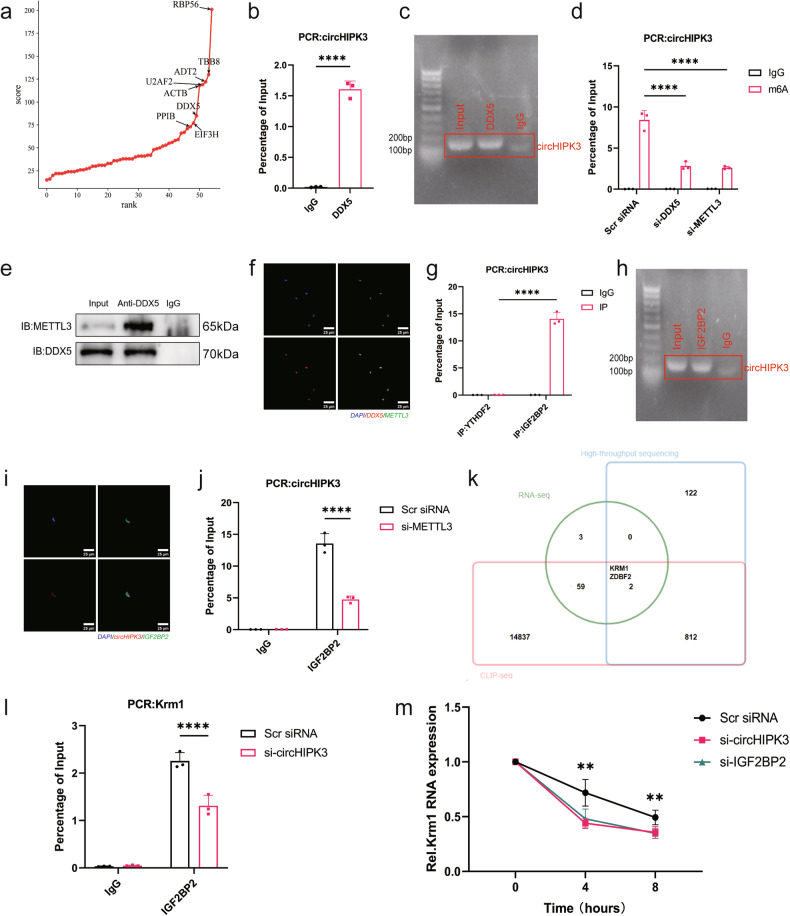
CircHIPK3 induces Krm1 mRNA stability in an m6A-dependent manner. **a** The proteins that bind to circHIPK3 were identified through mass spectrometry analysis and sorted by score, and the results revealed that DDX5 binds to circHIPK3. **b**, **c** RIP experiments were performed using anti-DDX5 or a negative control IgG antibody. The purified RNA was used for PCR to detect the amount of circHIPK3 (*n* = 3, two-tailed Student’s *t*-test). The right panel shows the results of agarose gel electrophoresis. **d** The m6A level of circHIPK3 was reduced after the knockdown of DDX5 and METTL3 (*n* = 3, ANOVA followed by a Bonferroni post hoc correction). **e**, **f** Immunofluorescence staining and co-IP experiments revealed the colocalization of DDX5 and METTL3 in AVICs (bar = 25 μm). **g**, **h** RIP experiments were performed using anti-IGF2BP2, anti-YTHDF2 or negative control IgG antibodies. The purified RNA was used for PCR to determine the amount of circHIPK3 (*n* = 3, ANOVA followed by a Bonferroni post hoc correction). IGF2BP2 had a greater affinity for circHIPK3 than did YTHDF2. **i** IF-FISH analysis showed that circHIPK3 colocalized with IGF2BP2 in the cytoplasm of AVICs (Pearson’s *r* = 0.92, bar =50 μm). **j** METTL3 knockdown decreased the binding of circHIPK3 to IGF2BP2 (*n* = 3, ANOVA followed by a Bonferroni post hoc correction). **k** Intersecting RNA-seq data with high-throughput sequencing and CLIP-seq data to screen for target genes. **l** The binding of IGF2BP2 to Krm1 mRNA was attenuated after circHIPK3 knockdown (*n* = 3, ANOVA followed by a Bonferroni post hoc correction). **m** Krm1 mRNA levels were determined in the AVICs treated with IGF2BP2 siRNA or circHIPK3 siRNA upon actinomycin D induction at the designated time points (*n* = 3, ANOVA followed by a Bonferroni post hoc correction). Asterisks represent statistically significant differences (**P < 0.01, ***P < 0.001, and ****P < 0.0001).

To determine how circHIPK3 regulates aortic valve calcification, we performed RNA pulldown experiments and analyzed immunoprecipitates from the pulldown experiments using mass spectrometry. Mass spectrometry revealed that circHIPK3 binds to DEAD-box helicase 5 (DDX5) (Fig. 4a), which drives N6-methyladenosine (m6A) modification of the target gene by recruiting methyltransferase 3 (METTL3)^23,24^. RNA immunoprecipitation (RIP) experiments and agarose gel electrophoresis demonstrated that DDX5 interacts with circHIPK3 (Fig. 4b, c). Furthermore, MeRIP-qPCR analysis confirmed that circHIPK3 was modified by m6A modification and that its m6A level decreased after METTL3 and DDX5 were knocked down (Fig. 4d). We predicted the specific m6A modification sites of circHIPK3 using SRAMP (http://www.cuilab.cn/sramp) and RMBase v2.0 (https://rna.sysu.edu.cn/rmbase/) and identified four high confidence sites on circHIPK3 (Supplementary Fig. 4a)^25,26^. The adenines of the predicted sites were all replaced with cytosines to construct the mutant circHIPK3 plasmid. MeRIP-qPCR experiments showed that the m6A modification of circHIPK3 was erased after transfection of the mutant circHIPK3 plasmid (Supplementary Fig. 4b). Immunofluorescence staining and coimmunoprecipitation (co-IP) experiments revealed that DDX5 colocalized with METTL3 in AVICs (Fig. 4e, f). These findings suggested that DDX5 is involved in the m6A modification of circHIPK3 in AVICs by recruiting METTL3.

We further explored the implications of the m6A modification of circHIPK3. Notably, m6A modification did not affect the expression or intracellular distribution of circHIPK3 (Supplementary Fig. 4c–e). Based on these results, we hypothesize that m6A-modified circHIPK3 may modulate the expression of its target genes rather than that of circHIPK3 itself. Notably, m6A-modified circRNA has been reported to increase mRNA stability by binding insulin-like growth factor 2 mRNA binding protein 2 (IGF2BP2), an m6A reader protein^14,15^. Therefore, we investigated whether circHIPK3 binds to IGF2BP2 via another m6A reader protein, YTH N6-methyladenosine RNA binding protein 2 (YTHDF2), which served as a control. RIP experiments showed that circHIPK3 interacted with IGF2BP2 but not with YTHDF2 (Fig. 4g, h). In addition, IF-FISH analysis revealed that circHIPK3 colocalized with IGF2BP2 in the cytoplasm of AVICs (Fig. 4i). The interaction between circHIPK3 and IGF2BP2 was strongly attenuated after METTL3 knockdown (Fig. 4j). This finding suggested that circHIPK3 interacted with IGF2BP2 in an m6A-dependent manner. Next, we searched for target mRNAs regulated by circHIPK3 and IGF2BP2. We performed RNA sequencing (RNA-seq) of circHIPK3-overexpressing AVICs (Supplementary Material 4, GSE254394) and compared the results with high-throughput sequencing data from aortic valves and CLIP-seq data for IGF2BP2 from starBase^27^ (Supplementary Fig. 4f, g, Fig. 4k). We selected the top-ranked Krm1 gene, whose expression is regulated by circHIPK3 and which can bind to IGF2BP2, as our target mRNA. Moreover, Krm1 expression was decreased in calcified aortic valves, which may be associated with CAVD progression. RIP experiments with an anti-IGF2BP2 antibody demonstrated that knockdown of circHIPK3 reduced Krm1 mRNA enrichment in AVICs (Fig. 4l). Moreover, knockdown of circHIPK3 significantly reduced Krm1 mRNA stability (Fig. 4m). Furthermore, we confirmed that circHIPK3 can regulate Krm1 expression (Supplementary Fig. 4h, i). These results suggested that m6A-modified circHIPK3 forms a complex with IGF2BP2 and Krm1 mRNA, which increases the stability of Krm1 mRNA and ultimately promotes Krm1 expression.

### Krm1 suppresses the osteogenic response of AVICs by inhibiting the Wnt/β-catenin pathway

**Fig. 5:**
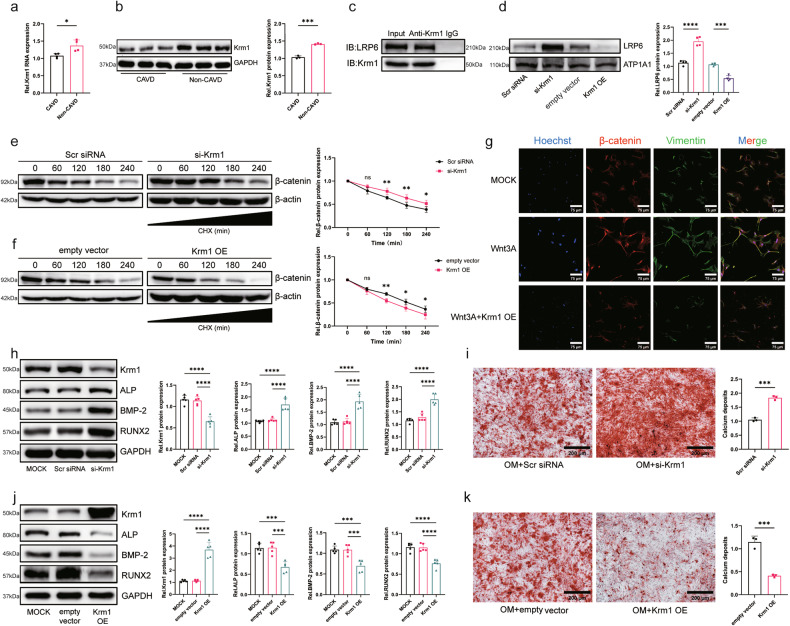
Krm1 inhibits the osteogenic response of AVICs by suppressing the Wnt/β-catenin pathway. **a**, **b** Krm1 expression in aortic valves was measured using qRT‒PCR and western blot assays (qRT‒PCR = 4. Western blot=3, two-tailed Student’s *t*-test). **c** A co-IP assay was used to identify the binding of Krm1 and LRP6 in AVICs. **d** LRP6 levels on membranes were determined by western blotting after Krm1 knockdown or overexpression (*n* = 4, ANOVA followed by a Bonferroni post hoc correction). **e**, **f** The stability of β-catenin was determined by performing a cycloheximide chase assay (*n* = 4, ANOVA followed by a Bonferroni post hoc correction). **g** Immunofluorescence staining showing the nuclear translocation of β-catenin (*n* = 3, bar = 100 μm). **h** The expression of osteogenic markers in AVICs after transfection with Scr siRNA or Krm1 siRNA was determined using western blot assays (*n* = 5, ANOVA followed by a Bonferroni post hoc correction). **i** Analysis of calcium deposition in the AVICs transfected with Scr siRNA or Krm1 siRNA using alizarin red S staining (*n* = 3, bar = 200 μm, two-tailed Student’s *t*-test). The red area represents calcium nodules. **j** The expression of osteogenic markers in AVICs after transfection with the vector or Krm1 overexpression vector was determined using western blot assays (*n* = 5, ANOVA followed by a Bonferroni post hoc correction). **k** Detection of calcium deposition in AVICs transfected with the vector or Krm1 overexpression vector using alizarin red S staining (*n* = 3, bar = 200 μm, two-tailed Student’s *t*-test). The red area represents the formation of calcium nodules. Asterisks represent statistically significant differences (**P < 0.01, ***P < 0.001, and ****P < 0.0001).

We first determined that Krm1 expression was reduced in calcified aortic valves, consistent with the sequencing results (Fig. 5a, b). Furthermore, we explored the mechanism by which Krm1 regulates aortic valve calcification. The STRING website showed that Krm1 binds to Dkks and LRP5/6 (Supplementary Fig. 5a). Previous studies have shown that Krm1 forms a ternary complex with Dkks and LRP5/6, promotes LRP5/6 removal from the membrane, and inhibits activation of the Wnt/β-catenin pathway^10^. Since LRP5 and LRP6 have similar effects, we selected LRP6, which is more highly expressed in the aortic valve, as our main subject of study. Co-IP experiments confirmed the binding of Krm1 to LRP6 (Fig. 5c). Furthermore, the amount of LRP6 on the cell membrane was inversely proportional to the expression of Krm1, indicating that the presence of Krm1 promoted LRP6 removal from the membrane (Fig. 5d). LRP6 acts as a membrane receptor that binds to Wnt ligands to promote downstream β-catenin stabilization and increase β-catenin intranuclear accumulation, which initiates the transcription of osteogenic genes^28^. A cycloheximide chase assay showed that Krm1 reduced the stability of β-catenin (Fig. 5e, f). Moreover, the nuclear translocation of β-catenin was induced by the Wnt pathway agonist recombinant Wnt3A, and this effect was counteracted by the overexpression of Krm1 (Fig. 5g). Next, we determined the effect of Krm1 on aortic valve calcification. Osteogenic markers, calcium deposition, and ALP activity were increased in AVICs after Krm1 knockdown (Fig. 5h, i, Supplementary Fig. 5b–e). However, overexpression of Krm1 reversed these effects (Fig. 5j, k, Supplementary Fig. 5f–i). Furthermore, treatment of AVICs with recombinant Wnt3A resulted in elevated expression of osteogenic markers, but overexpression of Krm1 abolished the stimulatory effect of Wnt3A (Supplementary Fig. 5j). These results suggested that Krm1 inhibited osteogenic responses in AVICs by inhibiting Wnt/β-catenin pathway activation.

### CircHIPK3 suppresses aortic valve calcification by inhibiting the Wnt/β-catenin pathway

**Fig. 6:**
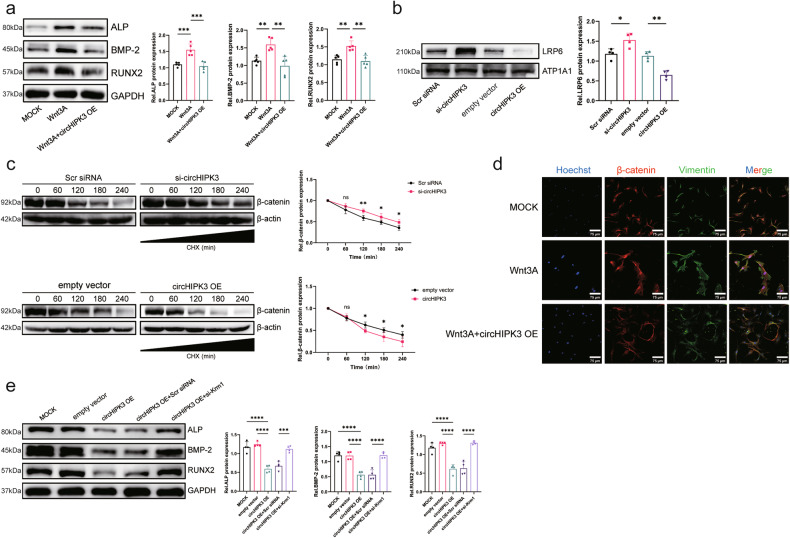
CircHIPK3 suppresses aortic valve calcification by inhibiting the Wnt/β-catenin pathway. **a** The expression of osteogenic markers was assessed using western blot assays (*n* = 5, ANOVA followed by a Bonferroni post hoc correction). **b** The content of LRP6 on the membrane was determined by performing a western blot analysis after knockdown or overexpression of circHIPK3 (*n* = 4, ANOVA followed by a Bonferroni post hoc correction). **c** The stability of β-catenin was determined by performing a cycloheximide chase assay (*n* = 4, ANOVA followed by a Bonferroni post hoc correction). **d** Immunofluorescence staining showing the nuclear translocation of β-catenin (*n* = 3, bar = 100 μm). **e** The expression of osteogenic markers was determined using western blot assays after overexpression of circHIPK3 and knockdown of Krm1 (*n* = 4, ANOVA followed by a Bonferroni post hoc correction). Asterisks represent statistically significant differences (**P < 0.01, ***P < 0.001, and ****P < 0.0001).

As described above, circHIPK3 regulates the expression of Krm1 in AVICs. In vivo, overexpression of circHIPK3 promoted the expression of Krm1 (Supplementary Fig. 6a, b). Then, we verified the effect of circHIPK3 on the Wnt/β-catenin pathway. After overexpression of circHIPK3, Wnt3A-mediated activation of the Wnt pathway, which promotes osteogenic responses in AVICs, was inhibited (Fig. 6a). Next, circHIPK3 reduced the amount of LRP6 on the membrane and decreased β-catenin stability to inhibit its nuclear translocation (Fig. 6b–d). Furthermore, overexpression of circHIPK3 inhibited the osteogenic response in AVICs, while knockdown of Krm1 counteracted this effect (Fig. 6e).

### Dkk2 collaborates with Krm1 to inhibit Wnt pathway activation

**Fig. 7:**
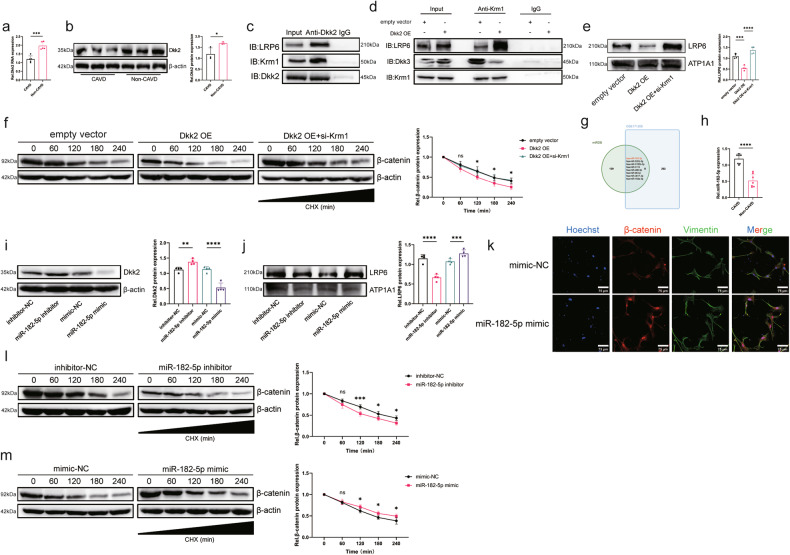
MiR-182-5p is involved in activating the Wnt/β-catenin pathway by suppressing Dkk2 expression. **a**, **b** The expression of Dkk2 in the aortic valve was determined using qRT‒PCR and western blot assays (qRT‒PCR = 5, western blot = 3, two-tailed Student’s *t*-test). **c** A co-IP assay was performed to observe the binding of Dkk2 to Krm1 and LRP6. **d** A co-IP assay was used to identify the interaction between Krm1 and LRP6 in AVICs after Dkk2 overexpression. **e** Determination of the LRP6 content on the membrane using western blotting (*n* = 4, ANOVA followed by a Bonferroni post hoc correction). **f** The stability of β-catenin was determined by performing a cycloheximide chase assay (*n* = 4, ANOVA followed by a Bonferroni post hoc correction). **g** Overlap of miRNA-seq (GSE171208) data with miRDB-predicted miRNAs binding Dkk2. The results revealed that miRNAs with increased expression in calcified valves may potentially target Dkk2. **h** The expression of miR-182-5p was measured using qRT‒PCR (*n* = 7). **i** The expression of Dkk2 in AVICs was determined by performing a western blot analysis after transfection with the miR-182-5p mimic or miR-182-5p inhibitor (qRT‒PCR = 4, western blot = 4, ANOVA followed by Bonferroni post hoc correction). **j** Determination of the LRP6 content on the membrane using western blotting (*n* = 4, ANOVA followed by a Bonferroni post hoc correction). **k** Immunofluorescence staining showing the nuclear translocation of β-catenin (*n* = 3, bar = 75 μm). **l**, **m** The stability of β-catenin was determined by performing a cycloheximide chase assay (*n* = 4, ANOVA followed by a Bonferroni post hoc correction). Asterisks represent statistically significant differences (**P < 0.01, ***P < 0.001, and ****P < 0.0001).

Krm1 inhibits the Wnt pathway in a mechanism requiring Dkks. We compared the expression of Dkks in aortic valves and found that Dkk4 was barely expressed and that Dkk1 was expressed minimally and not differentially, whereas Dkk2 was abundantly expressed and decreased in calcified aortic valves (Fig. 7a, b, Supplementary Fig. 7a). Therefore, we hypothesize that Dkk2 is the primary ligand that mediates Krm1-induced inhibition of Wnt signaling. Furthermore, co-IP experiments revealed that Dkk2 can bind Krm1 and LRP6 (Fig. 7c). Dkk3, another member of the Dkk family, appears to be an agonist of Wnt signaling that competes with Dkk1/2 for binding to Krm1. Overexpression of Dkk2 promoted the binding of Krm1 and LRP6 while reducing the binding of Krm1 and Dkk3 (Fig. 7d). However, Dkk2 knockdown had the opposite effect (Supplementary Fig. 7b). The amount of membrane-bound LRP6 and β-catenin stability were decreased after Dkk2 overexpression, but these effects were attenuated by Krm1 knockdown (Fig. 7e, f). Furthermore, Dkk2 inhibited the osteogenic response of AVICs, but Krm1 knockdown partially abolished the inhibitory effect of Dkk2 (Supplementary Fig. 8a–l, Supplementary Fig. 9a). These results suggest that Dkk2 inhibits the osteogenic response and Wnt/β-catenin pathway activity by promoting the Krm1-LRP6 interaction to form a ternary complex.

Next, we explored the mechanism by which Dkk2 expression is decreased in calcified aortic valves. Many studies have shown that Dkk2 is regulated by miRNAs^28,29^. Our previous study also demonstrated the important role of miRNAs in CAVD^30,31^. Therefore, we hypothesized that decreased expression of Dkk2 is associated with miRNAs. We used the miRDB database^32^ to predict miRNAs that bind Dkk2 and compared them with miRNAs that are differentially expressed in aortic valves^33^; among the intersecting miRNAs, miR-182-5p attracted our attention (Fig. 7g). Furthermore, miR-182-5p has been shown to activate the Wnt pathway^34,35^. First, we confirmed that miR-182-5p expression was elevated in calcified aortic valves (Fig. 7h). Then, a dual-luciferase assay revealed that miR-182-5p targets the 3’UTR of Dkk2 (Supplementary Fig. 9b, c). To determine the effect of miR-182-5p on Dkk2 expression and the Wnt pathway, we transfected AVICs with miR-182-5p mimic and miR-182-5p inhibitor. The western blot results showed that miR-182-5p inhibited Dkk2 expression and increased the amount of LRP6 on the membrane (Fig. 7i, j, Supplementary Fig. 9d). Moreover, miR-182-5p increased β-catenin stability and promoted its nuclear translocation (Fig. 7k–m). Notably, miR-182-5p promoted the expression of osteogenic markers but was counteracted by Dkk2 overexpression (Supplementary Fig. 9e). These results suggest that miR-182-5p is involved in activating the Wnt/β-catenin pathway by suppressing the expression of Dkk2.

## Discussion

CAVD is a public health problem, and its incidence increases with age^36^. To date, effective pharmacological interventions for CAVD are lacking, resulting in a very high disease burden. Therefore, identification of potential therapeutic targets for CAVD is urgently needed.

By comparing the circRNA expression profiles of human aortic valves, we discovered that circHIPK3 expression was decreased in calcified aortic valves. CircHIPK3 is highly conserved in humans, mice, and other mammals and is found in various organs, including the heart and lungs^20^. Previous studies have demonstrated the important role of circHIPK3 in cardiovascular disease^37^. Here, we observed that aortic valve stenosis was accompanied by thickening and calcium deposition in a CAVD mouse model. However, these changes were reversed after overexpression of circHIPK3 in vivo. The active osteogenic response in AVICs is a crucial factor in the progression of aortic valve calcification, and circHIPK3 significantly inhibits this response. Similarly, Zhang et al. demonstrated that circHIPK3 inhibits vascular calcification through the miR-106a-5p/MFN2 axis^21^. These findings demonstrate the ability of circHIPK3 to inhibit aortic valve calcification.

We further explored the specific mechanisms by which circHIPK3 regulates aortic valve calcification. As the most abundant RNA modification in eukaryotic cells, m6A can modulate RNA processes such as splicing, export, stability, translation, and degradation^13^. Wang et al. reported that m6A-modified DUSP26 is more stable than unmodified DUSP26, thereby affecting the expression of downstream DPP4 and the process of aortic valve calcification^38^. Recently, m6A modification has been recognized for its important biological function in regulating circRNAs^39^. Our results indicate that circHIPK3 is an m6A-modified circRNA, which is consistent with the findings of Zhou et al.^40^, and explain the mechanism by which m6A modification occurs. Numerous m6A readers have been identified, and recognition by different readers results in different regulatory outcomes of m6A modification. According to our findings, circHIPK3 has a greater affinity for IGF2BP2. m6A-modified circRNA was found to increase mRNA stability by functioning as a scaffold for the binding of IGF2BP2 and mRNA^14,15^. This phenomenon was also observed in the present study, and we identified Krm1 as a target gene of the circHIPK3/IGF2BP2 complex. Furthermore, knockdown of circHIPK3 reduced the binding of IGF2BP2 and Krm1 and decreased the stability of Krm1 mRNA. These results suggest that m6A-modified circHIPK3 is a key factor in promoting Krm1 mRNA stability.

Krm1 was originally described as a transmembrane protein containing the kringle domain, and recent reports have shown that it acts as a host cellular receptor to mediate SARS-CoV-2 entry^41^. Generally, Krm1 is described as a negative regulator of Wnt signaling. Wnt/β-catenin signaling plays key roles in bone development and homeostasis^42,43^ and is crucial for promoting aortic valve calcification^44^. Krm1 inhibits the Wnt pathway in a mechanism requiring Dkks. The Dkk family contains four members: Dkk1, Dkk2, Dkk3, and Dkk4. The presence of Dkk1/2 promotes Krm1 binding to LRP6. Dkks-Krm1-LRP6 form a ternary complex, which is removed from the cell membrane, thereby inhibiting Wnt signaling activity^10^. However, Dkk3 is a divergent member of the Dkk family that appears to be an agonist of Wnt signaling^45^. As shown by our high-throughput sequencing results, Dkk2 expression decreased only in the calcified aortic valve. Dkk2 has been reported to be involved in inhibiting various fibrotic processes^46^, and fibrosis is an early pathological feature of CAVD^47^. Recent studies have revealed that epithelial cell adhesion molecule (EpCAM) and Dkk2 compete to bind Krm1, reducing the Krm1-Dkk2 interaction, which prevents Krm1-Dkk2-mediated removal of LRP6 from the membrane and ultimately activates the Wnt pathway^48^. Our co-IP results showed that Dkk2 binds to Krm1 and LRP6 and reduces the amount of LRP6 on the cell membrane. Furthermore, overexpression of Dkk2 increased the binding of Krm1 to LRP6 while inhibiting the binding of Krm1 to Dkk3. Conversely, knockdown of Dkk2 led to an increase in Krm1-Dkk3 binding. These results suggest a regulatory pattern in which Krm1 inhibits Wnt pathway activation by forming a ternary complex with Dkk2 and LRP6 in normal aortic valves. However, in calcified aortic valves, decreased expression of Dkk2 led to increased Krm1-Dkk3 binding, resulting in LRP6 retention on the membrane to activate the Wnt pathway, ultimately exacerbating aortic valve calcification.

Since Krm1 expression is regulated by circHIPK3, we also wanted to identify the molecule that regulates the expression of Dkk2 in the aortic valve. Numerous miRNAs regulate Dkk2, promoting the cellular osteogenic response by suppressing its expression to activate the Wnt/β-catenin pathway^28,29^. In our previous studies and others, the regulatory role of miRNAs in CAVD was fully elucidated^28,29^. By comparing miRNA sequencing data from aortic valves and the miRNAs that bind Dkk2 determined with the miRDB database^32^, we identified miR-182-5p as the miRNA with the highest score. Although the relationship between miR-182-5p and Dkk2 has not been clarified, miR-182-5p has been reported to promote liver fibrosis^49^, in contrast to the ability of Dkk2 to inhibit fibrosis^46^. Surprisingly, miR-182-5p increased Wnt pathway activity in hepatocellular carcinoma by inhibiting P7TP3^34^ and FOXO3a^35^ expression. In the present study, we found that miR-182-5p expression was significantly upregulated in calcified aortic valves and that upregulated miR-182-5p expression mediated a decrease in Dkk2 expression. Moreover, miR-182-5p increased the amount of LRP6 on the membrane and the intranuclear accumulation of β-catenin. These results suggest that miR-182-5p is involved in Wnt pathway activation by targeting Dkk2.

In the present study, we identified a classic circRNA, circHIPK3, in human aortic valve tissues and determined its critical role in suppressing the development of aortic valve calcification. Specifically, circHIPK3 deficiency led to aortic valve calcification, whereas exogenous circHIPK3 supplementation slowed the progression of CAVD. Mechanistically, m6A-modified circHIPK3 promotes Krm1 expression to inhibit Wnt signaling activation. Furthermore, we found that miR-182-5p is involved in regulating Wnt signaling by inhibiting the expression of Dkk2, which is a ligand for Krm1. In conclusion, our study illustrates the important role of Krm1-Dkk2-mediated inhibition of Wnt signaling in the aortic valve, with noncoding RNAs acting as upstream factors to regulate their binding. Currently, the treatment of CAVD faces challenges, as statin drugs lack therapeutic benefits in CAVD^50^, further emphasizing the need to focus on valve-specific therapeutic targets. Our study highlights the importance of Wnt signaling and identifies its key factors. For the first time, we propose a role for Krm1 and Dkk2 in alleviating valvular calcification and introduce a model of dynamic binding between Krm1 and Dkk2/Dkk3, providing a deeper understanding of the role of Wnt signaling in aortic valve calcification. Targeting Wnt signaling may be a promising therapeutic strategy.

The present study has several limitations. First, our high-throughput sequencing was performed with a relatively small number of samples, which might have resulted in missing some important data. Second, we do not yet know the reason for the differences in circHIPK3 and miR-182-5p expression in different types of aortic valves, and further studies are still needed. Identifying key factors that regulate the expression of noncoding RNAs may be helpful for modulation of Wnt pathway activity and the progression of CAVD. Third, due to the lack of aortic valve-specific markers, AAV2-mediated circHIPK3 may not be specifically overexpressed in the aortic valve but instead may be overexpressed systemically. This phenomenon may lead to an unknown phenotype in the model, affecting the results.

### Supplementary information

Supplementary Material 1

Supplementary Material 2

Supplementary Material 3

Supplementary Material 4
