# Supplementary material for: Noncoding RNA regulates the expression of Krm1 and Dkk2 to synergistically affect aortic valve lesions
Source: Exp Mol Med. 2024 Jul 1;56(7):1560–73. doi: 10.1038/s12276-024-01256-5 (PMC11297286; doi:10.1038/s12276-024-01256-5)

Supplementary information

Supplementary Table 1. Demographic characteristics of controls and CAVD patients for this study

	Control (n=15)	CAVD (n=15)	*P*
Mean age (years)	59.1±4.78	62.2±6.3	NS
Male	15	15	NS
Weight (kg)	62.8±7.2	64.6±5.3	NS
Bicuspid valve	0	0	NS
Hypertension	8	10	NS
Atherosclerosis	3	4	NS
Diabetes mellitus	4	5	NS
COPD	0	0	NS

Note: Data were presented as mean ± standard deviation (SD) or %

Abbreviations: CAVD, calcific aortic valve disease; COPD, chronic obstructive pulmoriary disease; NS, nonsignificant.

Supplementary Table 2. Demographic characteristics of controls and CAVD animal models for this study

Injury animal models

	Sham	Injury+AAV2-Vector	Injury+AAV2-circHIPK3	*P*
Male	7	7	7	NS
Weight (g)	30.44±3.1	31.43±2.0	30.33±2.1	NS
Transvascular peak jet velocity	1.21±0.3	2.35±0.49	1.64±0.13	0.0002
Aortic valve area	1.21±0.23	0.63±0.13	0.92±0.14	0.0001

HFD-fed animal models

	ND	HFD+AAV2-Vector	HFD+AAV2-circHIPK3	*P*
Male	7	7	7	NS
Weight (g)	29.91±1.9	38.06±4.7	41.23±7.7	0.0007
Transvascular peak jet velocity	1.06±0.2	1.81±0.23	1.16±0.14	＜0.0001
Aortic valve area	1.26±0.15	0.79±0.17	1.09±0.11	0.0001

Supplementary Figure Legends

Supplementary Fig. 1: The intracellular distribution of circHIPK3. a The agarose gel image reveals that circHIPK3 is predominantly concentrated in the cytoplasm. GAPDH serves as a cytoplasmic marker, while U6 acts as a nuclear marker.

Supplementary Fig. 2: The role of circHIPK3 in the CAVD mouse model. a, b Adeno-associated virus subtype 2 expressing circHIPK3 (AAV2-circHIPK3) was injected into the CAVD mouse model (wire-injured C57BL/6 mice and HFD-fed $ApoE^{-/-}$ mice) to overexpress circHIPK3, while AAV2-expressing empty (AAV2-Vector) served as a control (n=7, Bar=75 μm). **c,d** Detection of the aortic valve area (AVA) in the CAVD mouse model (n=7, ANOVA followed by Bonferroni's post hoc test). Asterisks represent statistically significant differences (**$P < 0.01$, ***$P < 0.001$, and ****$P < 0.0001$).

Supplementary Fig. 3: Knockdown or overexpression of circHIPK3. a Small interfering RNA (siRNA) specifically targeting circHIPK3 but not L-HIPK3 (n=3, ANOVA followed by Bonferroni's post hoc test). The right panel shows results of agarose gel electrophoresis. **b** The control vector (circHIPK3 vector) or circHIPK3 overexpression vector (circHIPK3 OE) was transfected into AVICs, and the expression of circHIPK3 or L-HIPK3 was measured using qRT-PCR, which indicated that the circHIPK3 overexpression plasmid specifically amplified circHIPK3 without affecting L-HIPK3 expression (n=3, two-tailed Student's t-test). The right panel shows results of agarose gel electrophoresis.

Supplementary Fig. 4: The m6A modification did not affect the expression or

nucleocytoplasmic distribution of circHIPK3. a Potential m6A modification sites in circHIPK3. **b** MeRIP experiment to detect m6A levels of circHIPK3 after transfection of the mutant circHIPK3 plasmid (n=3, ANOVA followed by Bonferroni's post hoc test). **c** After the knockdown of DDX5 and METTL3, circHIPK3 expression was unchanged (n=3, ANOVA followed by Bonferroni's post hoc test). **d, e** qRT-PCR and immunofluorescence staining showed that knockdown of DDX5 and METTL3 did not affect the nucleocytoplasmic distribution of circHIPK3 (n=3, Bar=100 μm). **f** Perform RNA sequencing on AVICs post-transfection with control and circHIPK3 vectors, and generate a volcano plot from the differential expression gene (n=4). **g** High-throughput results showed decreased Krm1 expression in calcified aortic valves. **h, i** Krm1 expression was detected using western blot assays after the knockdown or overexpression of circHIPK3 (n=3, ANOVA followed by Bonferroni's post hoc test).Asterisks represent statistically significant differences (**$P < 0.01$, ***$P < 0.001$, and ****$P < 0.0001$).

Supplementary Fig. 5: Krm1 inhibits the osteogenic response of AVICs. a The STRING website created a protein interaction network for Krm1 based on the reliability of the evidence, and LRP6 is included in this interaction network. **b, c** A Krm1 siRNA (si-Krm1) was used to knock down Krm1 expression in AVICs (n=3, ANOVA followed by Bonferroni's post hoc test). **d** The ALP activity of AVICs treated with the Krm1 siRNA was detected (n=3, Bar=200 μm, two-tailed Student's t-test). **e** Immunofluorescence staining was used to detect osteocalcin expression in AVICs transfected with the Krm1 siRNA (n=3, Bar=50 μm, ANOVA followed by

Bonferroni's post hoc test). **f, g** The Krm1 overexpression vector (Krm1 OE) successfully induced Krm1 overexpression in AVICs (n=3, ANOVA followed by Bonferroni's post hoc test). **h** ALP activity in AVICs treated with the Krm1 overexpression vector was detected (n=3, Bar=200 μm, two-tailed Student's t-test). **i** Immunofluorescence staining was used to detect osteocalcin expression in AVICs transfected with the Krm1 overexpression vector (n=3, Bar=50 μm, ANOVA followed by Bonferroni's post hoc test). **j** The expression of osteogenic markers in AVICs after treatment with recombinant Wnt3A and Krm1 overexpression vector was detected using western blot assays (n=5, ANOVA followed by Bonferroni's post hoc test). Asterisks represent statistically significant differences (**$P < 0.01$, ***$P < 0.001$, and ****$P < 0.0001$).

Supplementary Fig. 6: CircHIPK3 upregulates the expression of Dkk2 *in vivo*. a, b Detecting the expression of Krm1 in different groups using immunofluorescence. (n=7, ANOVA followed by Bonferroni's post hoc test). Asterisks represent statistically significant differences (**$P < 0.01$, ***$P < 0.001$, and ****$P < 0.0001$).

Supplementary Fig. 7:The expression pattern of Dkk2 in AVICs. a Mapping Dkks expression based on high-throughput sequencing results. Among them, Dkk2 had the highest expression level, which was significantly decreased in calcified aortic valve tissues. Dkk4 had less expression and was not significantly different. Dkk1 was essentially unexpressed. **b** A co-IP assay detected the interaction of Krm1 and LRP6 in AVICs after Dkk2 knockdown.

Supplementary Fig. 8: Dkk2 inhibits the osteogenic response of AVICs. a, b A

Dkk2 siRNA (si-Dkk2) was used to knock down Dkk2 expression in AVICs (n=3, ANOVA followed by Bonferroni's post hoc test). **c** The expression of osteogenic markers in AVICs after transfection with the Scr siRNA or Dkk2 siRNA was detected using western blot assays (n=6, ANOVA followed by Bonferroni's post hoc test). **d** Immunofluorescence staining was used to detect osteocalcin expression in AVICs transfected with the Dkk2 siRNA (n=3, Bar=50 μm, ANOVA followed by Bonferroni's post hoc test). **e** Detection of calcium deposition in AVICs transfected with the Scr siRNA or Dkk2 siRNA using alizarin red S staining (n=3, Bar=200 μm, two-tailed Student's t-test). **f** The ALP activity of AVICs treated with the Dkk2 siRNA was detected (n=3, Bar=200 μm, two-tailed Student's t-test). **g, h** The Dkk2 overexpression vector (Dkk2 OE) successfully induced Dkk2 overexpression in AVICs (n=3, ANOVA followed by Bonferroni's post hoc test). **i** The expression of osteogenic markers in AVICs after transfection with the vector or Dkk2 overexpression vector was detected using western blot assays (n=6, ANOVA followed by Bonferroni's post hoc test). **j** Immunofluorescence staining was used to detect osteocalcin expression in AVICs transfected with the Dkk2 overexpression vector (n=3, Bar=50 μm, ANOVA followed by Bonferroni's post hoc test). **k** Detection of calcium deposition in AVICs transfected with the vector or Dkk2 overexpression vector using alizarin red S staining (n=3, Bar=200 μm, two-tailed Student's t-test). **l** ALP activity in AVICs treated with the Dkk2 overexpression vector was detected (n=3, Bar=200 μm, two-tailed Student's t-test). Asterisks represent statistically significant differences (**$P < 0.01$, ***$P < 0.001$, and ****$P < 0.0001$).

Supplementary Fig. 9: miR-182-5p regulates the expression of Dkk2. a The expression of osteogenic markers in AVICs was detected by performing a western blot assay (n=4, ANOVA followed by Bonferroni's post hoc test). **b** Potential binding sites for miR-182-5p in Dkk2. **c** Relative luciferase activity was determined by performing dual-luciferase assays (n=3, ANOVA followed by Bonferroni's post hoc test). **d** The expression of Dkk2 in AVICs was determined by performing qRT-PCR after transfection with the miR-182-5p mimic or miR-182-5p inhibitor (qRT-PCR=4, Western blot=4, ANOVA followed by Bonferroni's post hoc test). **e** The expression of osteogenic markers in AVICs was detected by performing a western blot assay (n=4, ANOVA followed by Bonferroni's post hoc test).Asterisks represent statistically significant differences (**$P < 0.01$, ***$P < 0.001$, and ****$P < 0.0001$).

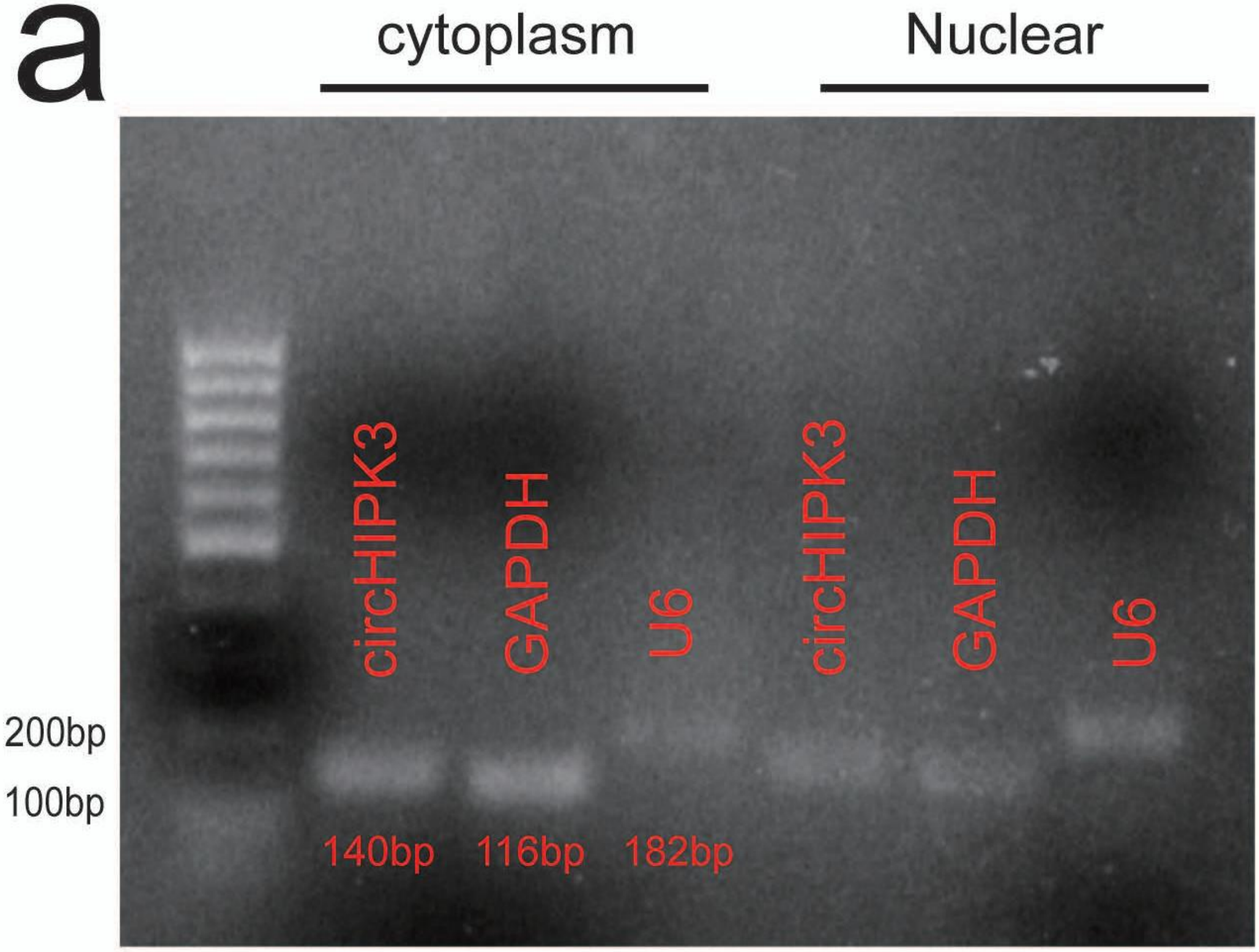
a
cytoplasm
Nuclear
circHIPK3
GAPDH
U6
circHIPK3
GAPDH
U6
200bp
100bp
140bp
116bp
182bp

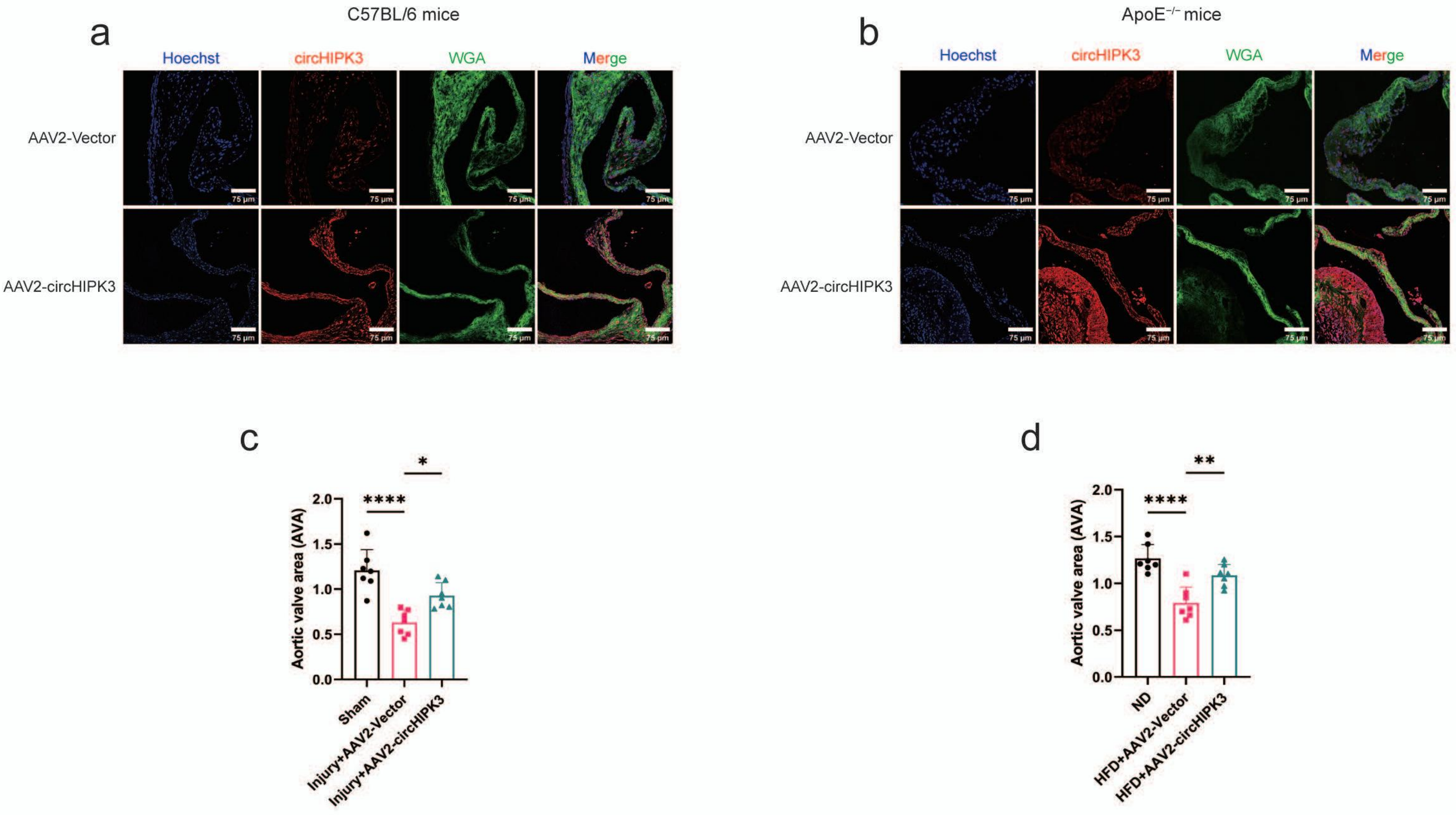

C57BL/6 mice
a
Hoechst
circHIPK3
WGA
Merge
AAV2-Vector
AAV2-circHIPK3
ApoE−/− mice
b
c
Aortic valve area (AVA)
Sham
Injury+AAV2-Vector
Injury+AAV2-circHIPK3
d
ND
HFD+AAV2-Vector
HFD+AAV2-circHIPK3

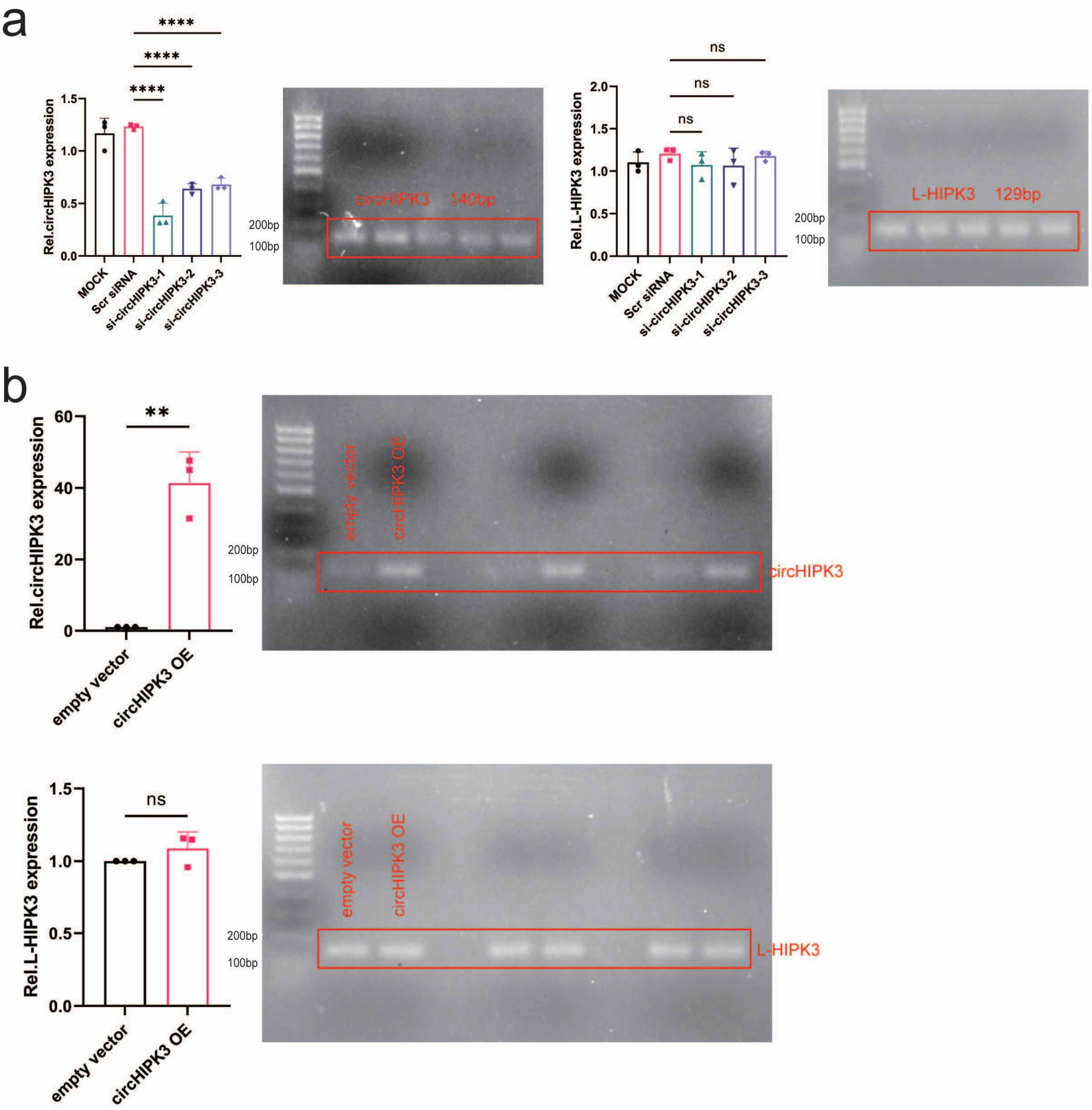

a
****
****
****
Rel.circHIPK3 expression
1.5
1.0
0.5
0.0
MOCK
Scr siRNA
si-circHIPK3-1
si-circHIPK3-2
si-circHIPK3-3
circHIPK3
140bp
200bp
100bp
ns
ns
ns
Rel.L-HIPK3 expression
2.0
1.5
1.0
0.5
0.0
MOCK
Scr siRNA
si-circHIPK3-1
si-circHIPK3-2
si-circHIPK3-3
L-HIPK3
129bp
200bp
100bp
b
**
Rel.circHIPK3 expression
60
40
20
0
empty vector
circHIPK3 OE
empty vector
circHIPK3 OE
200bp
100bp
circHIPK3
ns
Rel.L-HIPK3 expression
1.5
1.0
0.5
0.0
empty vector
circHIPK3 OE
empty vector
circHIPK3 OE
200bp
100bp
L-HIPK3

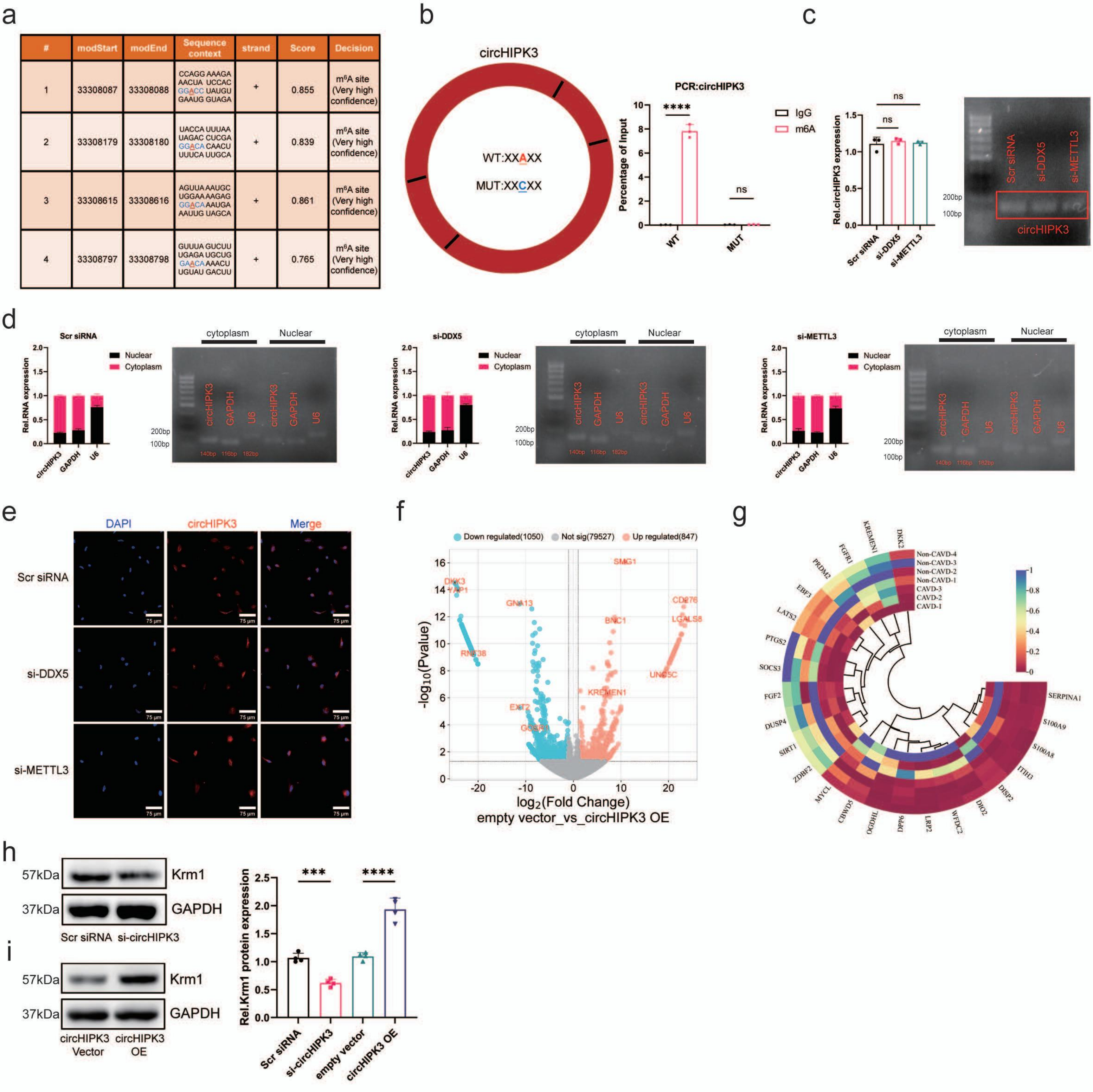
a
#
modStart
modEnd
Sequence context
strand
Score
Decision
1
33308087
33308088
CCAGG AAAGA AACUA UCCAC GGACC UAUGU GAAUG GUAGA
+
0.855
m6A site (Very high confidence)
2
33308179
33308180
UACCA UUUAA UAGAC CUCGA GGACA CAACU UUUCA UUGCA
+
0.839
m6A site (Very high confidence)
3
33308615
33308616
AGUUA AAUGC UGGAA AAGAG GGACA AAUGA AAUUG UAGCA
+
0.861
m6A site (Very high confidence)
4
33308797
33308798
GUUUA GUCUU UGAGA UGCUG GAACA AAACU UGUAU GACUU
+
0.765
m6A site (Very high confidence)
b
circHIPK3
WT:XXAXX
MUT:XXCXX
PCR:circHIPK3
IgG
m6A
Percentage of Input
****
ns
WT
MUT
c
ns
ns
Rel.circHIPK3 expression
Scr siRNA
si-DDX5
si-METTL3
200bp
100bp
circHIPK3
d
Scr siRNA
si-DDX5
si-METTL3
Nuclear
Cytoplasm
Rel.RNA expression
circHIPK3
GAPDH
U6
cytoplasm
Nuclear
200bp
100bp
140bp 116bp 182bp
e
DAPI
circHIPK3
Merge
Scr siRNA
si-DDX5
si-METTL3
75 μm
f
Down regulated(1050)
Not sig(79527)
Up regulated(847)
-log10(Pvalue)
log2(Fold Change)
empty vector_vs_circHIPK3 OE
DKK3
SMG1
GNA13
CD276
BNC1
LGALS8
RNF38
UNC5C
KREMEN1
EXT2
g
Non-CAVD-4
Non-CAVD-3
Non-CAVD-2
Non-CAVD-1
CAVD-3
CAVD-2
CAVD-1
DKK2
KREMEN1
FGFR1
PRDM2
EBF3
LATS2
PTGS2
SOCS3
FGF2
DUSP4
SIRT1
ZDBF2
MYCL
CBWD5
OGDHL
DPP6
LRP2
WFDC2
DIO2
DISP2
ITIH3
S100A8
S100A9
SERPINA1
h
57kDa
Krm1
37kDa
GAPDH
Scr siRNA
si-circHIPK3
i
57kDa
Krm1
37kDa
GAPDH
circHIPK3 Vector
circHIPK3 OE
***
****
Rel.Krm1 protein expression
Scr siRNA
si-circHIPK3
empty vector
circHIPK3 OE

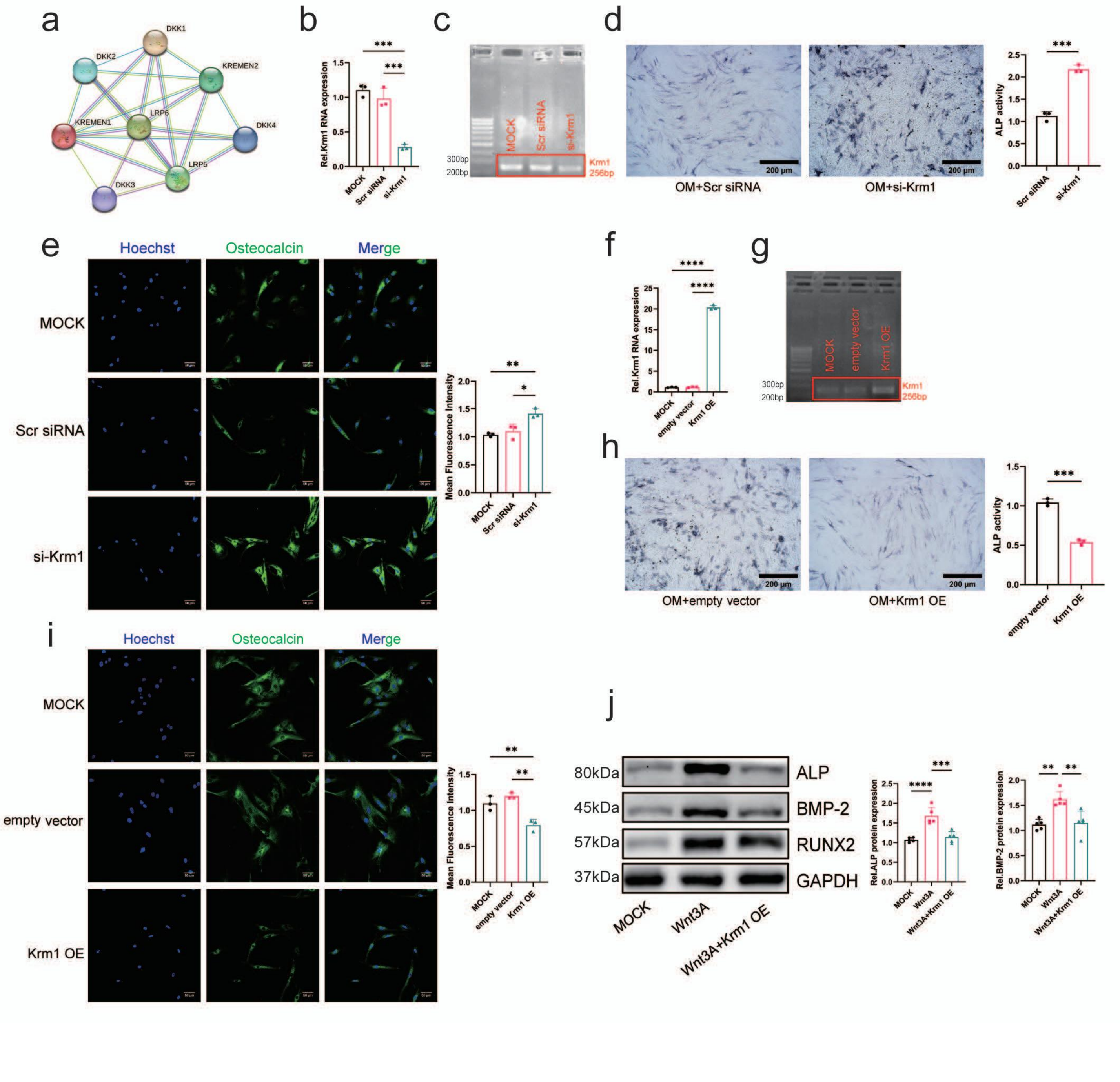

a
b
c
d
e
f
g
h
i
j
DKK1
DKK2
KREMEN2
LRP6
KREMEN1
DKK4
LRP5
DKK3
Rel.Krm1 RNA expression
MOCK
Scr siRNA
si-Krm1
300bp
200bp
Krm1
256bp
OM+Scr siRNA
OM+si-Krm1
200 μm
ALP activity
Hoechst
Osteocalcin
Merge
Mean Fluorescence Intensity
empty vector
Krm1 OE
OM+empty vector
OM+Krm1 OE
80kDa
45kDa
57kDa
37kDa
ALP
BMP-2
RUNX2
GAPDH
Wnt3A
Wnt3A+Krm1 OE
Rel.ALP protein expression
Rel.BMP-2 protein expression
Rel.RUNX2 protein expression

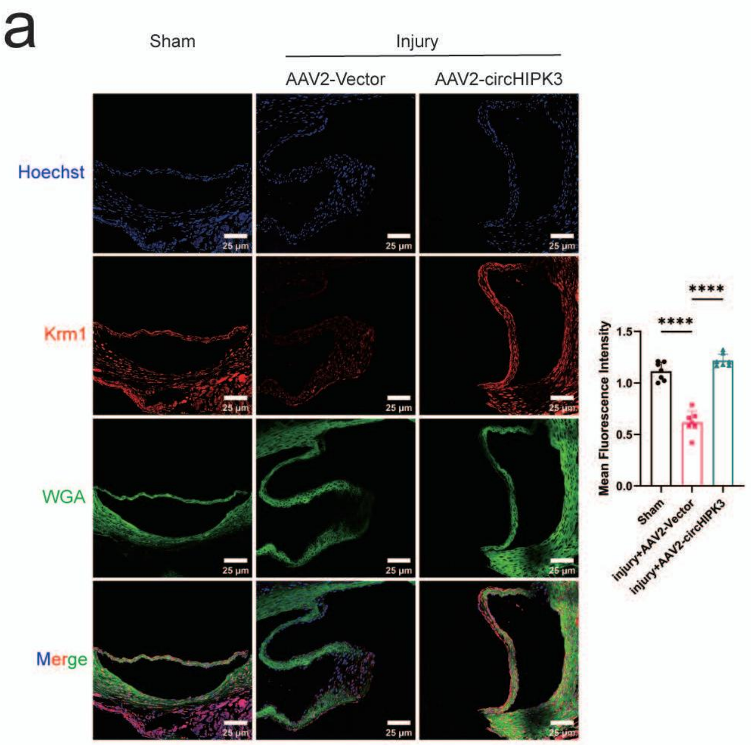
a
Sham
Injury
AAV2-Vector
AAV2-circHIPK3
Hoechst
Krm1
WGA
Merge
25 μm
Mean Fluorescence Intensity
1.5
1.0
0.5
0.0
****
****
Sham
injury+AAV2-Vector
injury+AAV2-circHIPK3

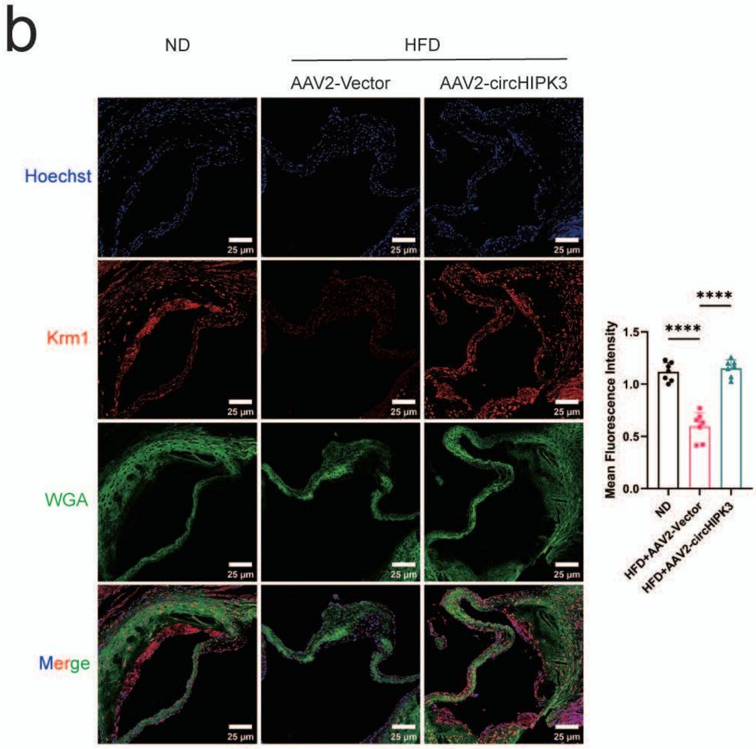
b
ND
HFD
AAV2-Vector
AAV2-circHIPK3
Hoechst
Krm1
WGA
Merge
25 μm
Mean Fluorescence Intensity
1.5
1.0
0.5
0.0
****
****
ND
HFD+AAV2-Vector
HFD+AAV2-circHIPK3

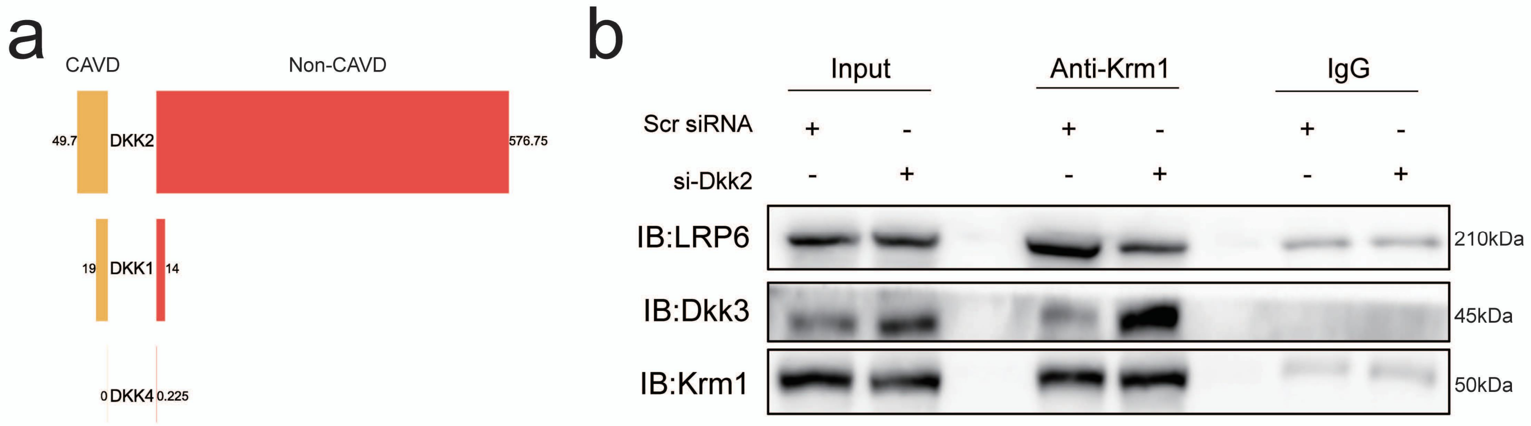

a
CAVD
Non-CAVD
49.7
DKK2
576.75
19
DKK1
14
0
DKK4
0.225
b
Input
Anti-Krm1
IgG
Scr siRNA
si-Dkk2
IB:LRP6
IB:Dkk3
IB:Krm1
210kDa
45kDa
50kDa

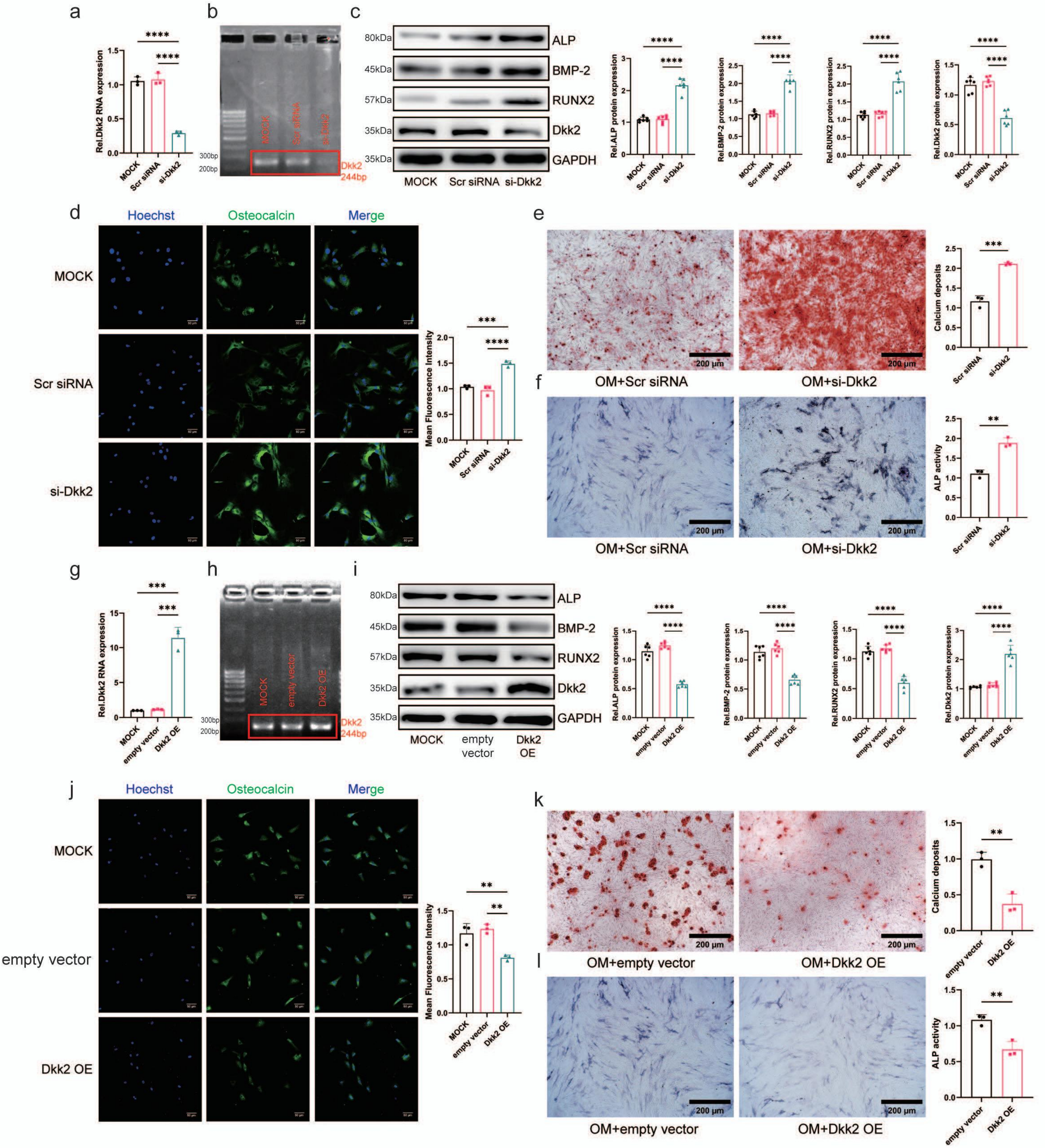
a
b
c
Rel.Dkk2 RNA expression
MOCK
Scr siRNA
si-Dkk2
300bp
200bp
Dkk2
244bp
80kDa
45kDa
57kDa
35kDa
ALP
BMP-2
RUNX2
Dkk2
GAPDH
Rel.ALP protein expression
Rel.BMP-2 protein expression
Rel.RUNX2 protein expression
Rel.Dkk2 protein expression
d
Hoechst
Osteocalcin
Merge
Mean Fluorescence Intensity
e
f
Calcium deposits
ALP activity
200 μm
OM+Scr siRNA
OM+si-Dkk2
g
h
i
empty vector
Dkk2 OE
j
k
l
OM+empty vector
OM+Dkk2 OE

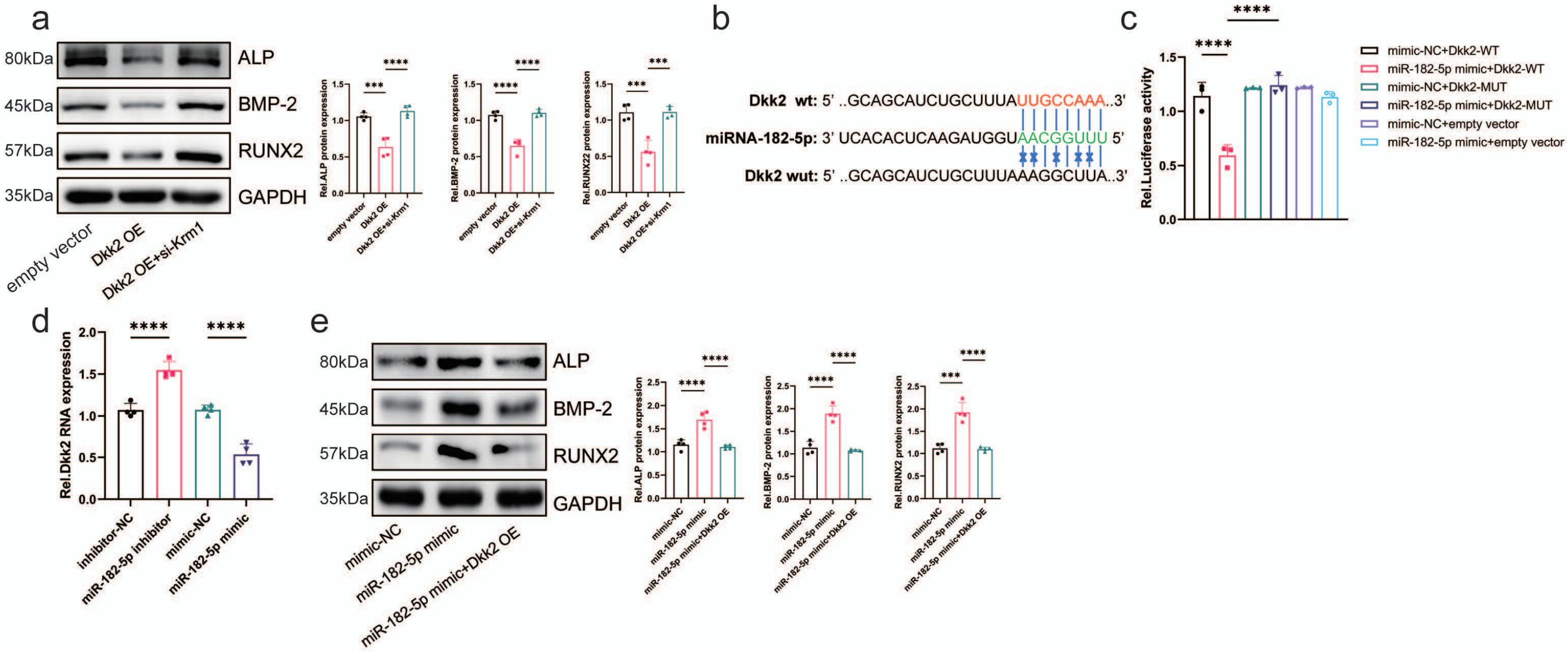
a
80kDa
45kDa
57kDa
35kDa
ALP
BMP-2
RUNX2
GAPDH
empty vector
Dkk2 OE
Dkk2 OE+si-Krm1
Rel.ALP protein expression
Rel.BMP-2 protein expression
Rel.RUNX22 protein expression
b
Dkk2 wt: 5' ..GCAGCAUCUGCUUUAUUGCCAAA..3'
miRNA-182-5p: 3' UCACACUCAAGAUGGUAACGGUUU 5'
Dkk2 wut: 5' ..GCAGCAUCUGCUUUAAAGGCUUA..3'
c
Rel.Luciferase activity
mimic-NC+Dkk2-WT
miR-182-5p mimic+Dkk2-WT
mimic-NC+Dkk2-MUT
miR-182-5p mimic+Dkk2-MUT
mimic-NC+empty vector
miR-182-5p mimic+empty vector
d
Rel.Dkk2 RNA expression
inhibitor-NC
miR-182-5p inhibitor
mimic-NC
miR-182-5p mimic
e
mimic-NC
miR-182-5p mimic
miR-182-5p mimic+Dkk2 OE
Rel.ALP protein expression
Rel.BMP-2 protein expression
Rel.RUNX2 protein expression

PCR primer sequence and siRNA sequence

Primer sequence		
circHIPK3	Forward	5’-TATGTTGGTGGATCCTGTTCGGCA-3’
	Reverse	5’-TGGTGGGTAGACCAAGACTTGTGA-3’
HIPK3	Forward	5’-TCACAAGTCTTGGTCTACCCA-3’
	Reverse	5’-CACATAGGTCCGTGGATAGTTTC-3’
GAPDH	Forward	5’-TGTGGGCATCAATGGATTTGG-3’
	Reverse	5’-ACACCATGTATTCCGGGTCAAT-3’
U6	Forward	5’-AGTAAGCCCTTGCTGTCAGTG-3’
	Reverse	5’-CCTGGGTCTGATAATGCTGGG-3’
Krm1	Forward	5’-GAGGCAGCCAGTACCGAATG-3’
	Reverse	5’-CCGAGTCCCTGATGTCAAATAG-3’
Dkk2	Forward	5’-AGTACCCGCTGCAATAATGG-3’
	Reverse	5’-GAAATGACGAGCACAGCAAA-3’
miR-182-5p	Forward	5’-GGCAATGGTAGAACTCACAC-3’
**siRNA sequence**		
si-circHIPK3-1		5’-UCUCGCUACUACAGGUAUG-3′
si-circHIPK3-2		5’-AAUCUCGGUACUACAGGUAUG-3′
si-circHIPK3-3		5’-UACAGGUAUGGCCUCACAAGU-3′
si-DDX5		121480, siRNA, Thermo Scientific
si-METTL3		132906, siRNA, Thermo Scientific
si-YTHDF2		122894, siRNA, Thermo Scientific

si-Krm1	103954, siRNA, Thermo Scientific
si-Dkk2	134328, siRNA, Thermo Scientific

siRNAs target sites

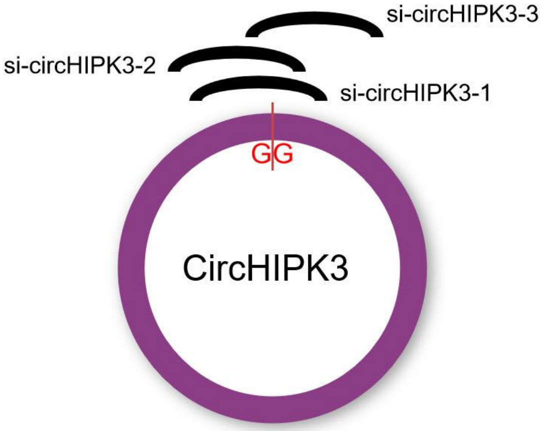

si-circHIPK3-1	5′-UCUCGCUACUACAGGUAUG-3′
circHIPK3 junction	5′-TCTACAATCTCGGTACTACAGGTATGGCCTCACAAGTCTT-3′
si-circHIPK3-2	5′-AAUCUCGGUACUACAGGUAUG-3′
si-circHIPK3-3	5′-UACAGGUAUGGCCUCACAAGU-3′

vector structure

The AAV2-circHIPK3 used in the study was provided by Vigene (Shandong, China), and the vector structure diagram is as follows:

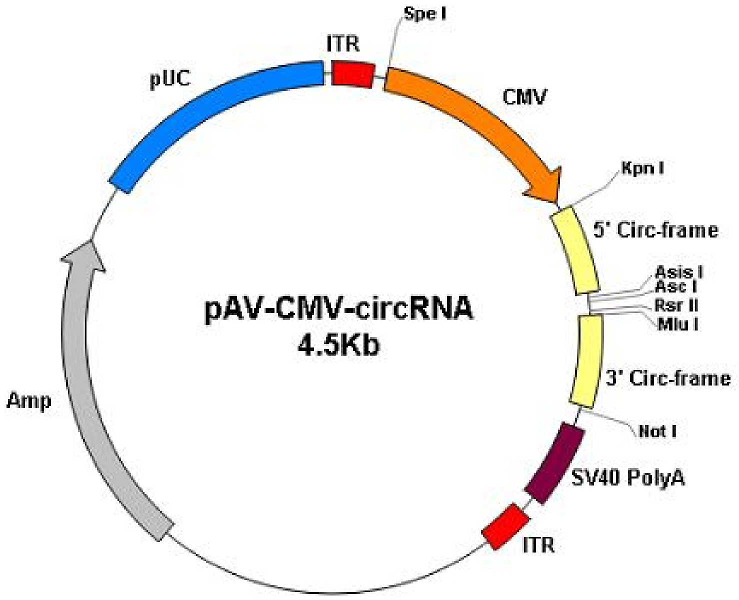

The circHIPK3 overexpression plasmid used in the study was provided by GeneSeed (Guangzhou, China), and the vector structure diagram is as follows:

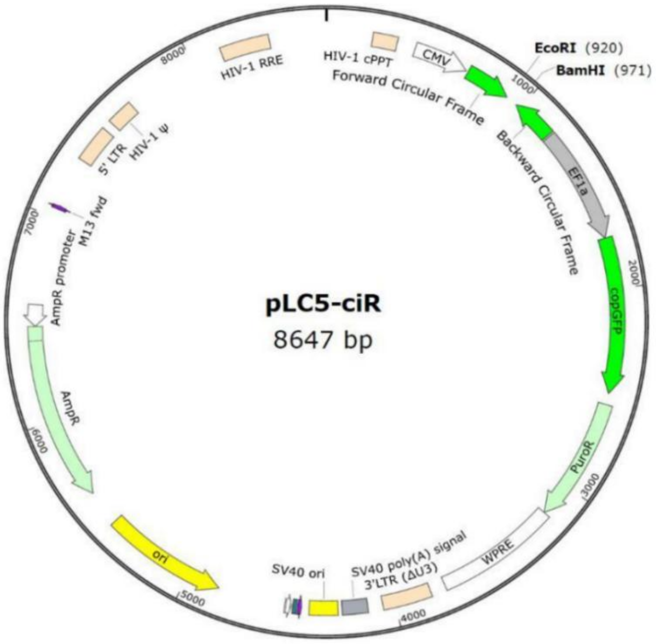

The size of the empty vector is 8647 bp, and the size of the circHIPK3 overexpression vector is 9701 bp.

The Krm1 and Dkk2 overexpression plasmids used in the study were provided by GeneChem (Shanghai, China), and the vector structure diagram is as follows:

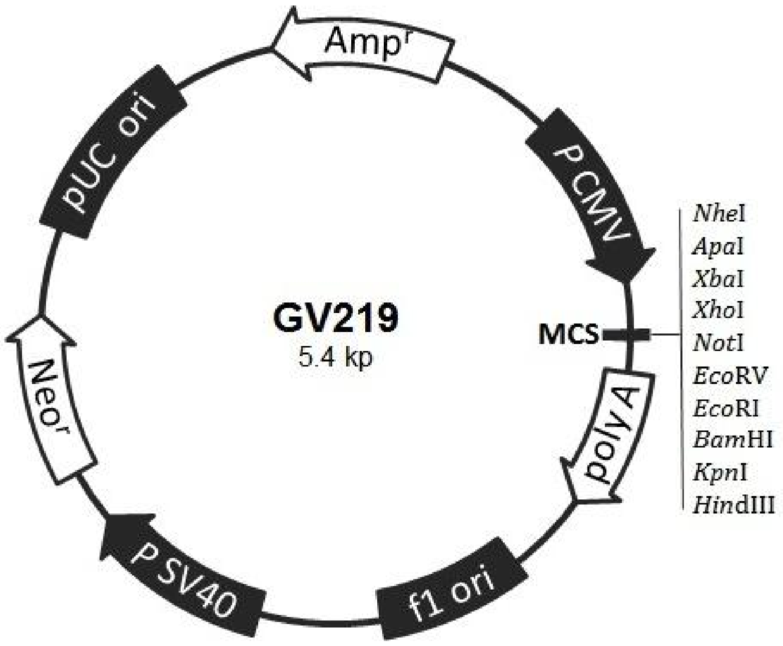

Supplement: Supplementary file 1 — Supplementary Material 1 [file 12276_2024_1256_MOESM1_ESM.pdf]
